# Operating-Room scheduling under uncertainty: balancing elective and emergency surgeries

**DOI:** 10.1007/s10729-026-09791-5

**Published:** 2026-09-10

**Authors:** Hajar Sadegh Zadeh, Lele Zhang, Mark Fackrell, Hamideh Anjomshoa

**Affiliations:** 1 ARC Training Centre in Optimisation Technologies, Integrated Methodologies, and Applications (OPTIMA), Melbourne, Australia; 2 School of Mathematics and Statistics, The University of Melbourne, Melbourne, Australia

**Keywords:** Operating room scheduling, Stochastic optimisation, Emergency case management, Elective surgery scheduling, Uncertainty management

## Abstract

Balancing elective and emergency surgeries in operating room (OR) scheduling is challenging due to uncertainty in surgery durations and emergency arrivals. Traditional approaches, such as dedicated OR allocation and the Break-in-Moment (BIM) policy, can result in underutilised capacity and high emergency cancellation rates. This paper proposes a two-stage stochastic optimisation model that incorporates endogenous buffer allocation within the OR schedule. Rather than relying on policy-based or heuristically defined buffer times, the proposed framework determines the timing and placement of buffer slots as part of the optimisation process. In the first stage, elective surgeries and buffer placements are jointly determined at the beginning of the week. In the second stage, OR allocations are adjusted in response to realised emergency arrivals and surgery-duration deviations. The model is solved using the Sample Average Approximation (SAA) method and benchmarked against deterministic, perfect-information, and robust optimisation approaches. We validate the model using a synthetic dataset calibrated to real OR data from a public hospital. Computational experiments show that the proposed endogenous buffer-allocation approach achieves significant cost and cancellation reductions compared with all benchmark methods, while closely approaching the idealised perfect-information solution. Results over a six-month period indicate an 18% reduction in total weekly costs, an increase in average OR utilisation from 78% to 84%, and a substantial decrease in emergency cancellations. These findings demonstrate the value of endogenous buffer allocation for improving OR efficiency and responsiveness under uncertainty.

## Introduction

Hospitals around the world are under growing pressure to provide high-quality care with limited resources [1]. Operating Rooms (ORs) are central to these challenges, consuming substantial resources and shaping overall patient flow [2]. Effective OR scheduling is therefore essential for maximising facility utilisation, reducing patient wait times, and minimising costs and cancellations. Yet, scheduling is complicated by two major factors. First, surgeries have inherently uncertain durations, making accurate planning challenging. Additionally, emergency case arrivals are uncertain and must often share OR capacity with elective procedure, adding complexity. Together, these uncertainties create a dynamic environment demanding robust, adaptable scheduling practices.

A common approach assigns dedicated OR blocks exclusively to elective or emergency procedures. While this prevents emergency cases from displacing elective ones, it often results in underuse of OR capacity when emergency demand is low. Conversely, a surge in emergency arrivals can severely disrupt elective procedures, causing cancellations or delays. To address these issues, some hospitals use a Break-in-Moment (BIM) policy, inserting emergency cases into the first available OR [3]. Others employ policy-based buffer times between surgeries. However, BIM becomes ineffective when scheduled surgeries run long, jeopardising urgent patient care. Policy-based buffer times often rely on deterministic assumptions that fail to capture real-world variability, limiting their effectiveness. Moreover, hospitals are hesitant to blend elective and emergency surgeries in the same ORs due to coordination complexities.

This paper tackles these challenges by introducing a two-stage stochastic optimisation model that strategically incorporates flexible buffer times into the operating-room schedule. Rather than using policy-based buffer buffer durations or heuristic insertion rules, the proposed model determines when and where buffer times should be placed across operating-room blocks before uncertainty is realised. These buffer placements are determined while accounting for uncertainty in emergency arrivals and surgery durations.

In the first stage, elective surgeries are scheduled at the beginning of the week while reserving buffer capacities based on anticipated emergency demand. In the second stage, the schedule is adjusted according to realised emergency arrivals and surgery durations through scenario-based recourse decisions. Unlike approaches that rely solely on BIM policies, dedicated emergency ORs, or policy-based buffer rules, the proposed framework uses uncertainty to guide where buffer capacity should be reserved so that the schedule can better balance OR utilisation, overtime, and cancellation risk.

To solve the model, we employ the Sample Average Approximation (SAA) technique, which efficiently estimates the expected recourse cost by sampling multiple realisations of uncertainty and transforming the stochastic formulation into a deterministic equivalent. In addition, we consider a robust (min–max) optimisation benchmark, inspired by the approach of Neyshabouri and Berg [4], solved via the Column-and-Constraint Generation (CCG) method, to provide a comparative baseline and evaluate the conservativeness of worst-case decision-making. The two-stage stochastic model is further compared against deterministic expected-value and perfect-information benchmarks, and validated using a synthetic dataset calibrated to real hospital data. Computational experiments confirm that the proposed model achieves strong improvements in OR utilisation and cost reduction while maintaining tractable solution times across hundreds of daily instances.

Our main contribution is the development of an endogenous and uncertainty-informed buffer allocation mechanism within a two-stage stochastic optimisation framework for operating-room scheduling. Rather than treating buffer time as a policy-based input or a heuristic rule, the proposed model determines the timing and placement of buffer slots as part of the optimisation process through the first-stage decision variable $h_{bt}$. This allows buffer allocation to vary across surgical blocks, weekdays, and specialties according to the expected impact of emergency arrivals and surgery-duration uncertainty.

This distinction is important because policy-based buffer allocations can be inefficient in practice: overly conservative buffers reduce OR utilisation, while insufficient buffers increase the likelihood of emergency or elective cancellations. By embedding buffer placement within the stochastic optimisation model, the proposed framework explicitly balances these competing risks and produces schedules that are both responsive and cost-efficient. In this way, the contribution of this study lies not merely in considering stochastic emergency arrivals and surgery durations, but in using these uncertainties to shape preventive scheduling decisions through endogenous buffer placement.

From a practical perspective, the model is evaluated using a synthetic dataset calibrated to real data from a major Australian hospital. The computational results show that the proposed endogenous buffer-allocation approach improves OR utilisation and reduces cancellations compared with current practice and alternative deterministic, perfect-information, and robust optimisation benchmarks.

The remainder of this paper is structured as follows. Section 2 presents the motivation behind this study and discusses the practical setting. Section 3 reviews the literature on OR scheduling under uncertainty. Section 4 outlines our modeling approach and methodological framework. Section 5 describes the computational experiments and solution methods. Section 6 details the numerical results and analysis. Finally, Section 7 concludes the paper and suggests directions for future research.

## Motivation

**Fig. 1:**
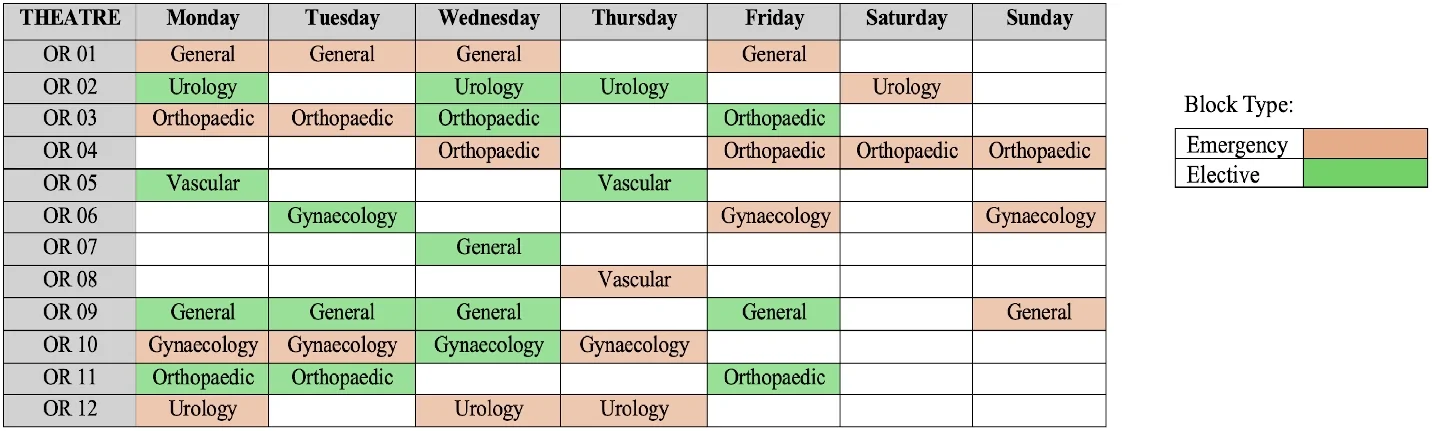
Master surgery schedule (May to Nov 2019)

We analysed surgical data from an Australian hospital with 12 ORs covering multiple specialties such as general surgery, orthopaedics, and gastroenterology. Figure 1 presents the Master Surgery Schedule (MSS) used from May to November 2019, where each cell represents a surgical block—a combination of an OR, a weekday, and a specialty. Surgical blocks are assigned to either elective or emergency cases, following a rigid allocation system.

**Fig. 2:**
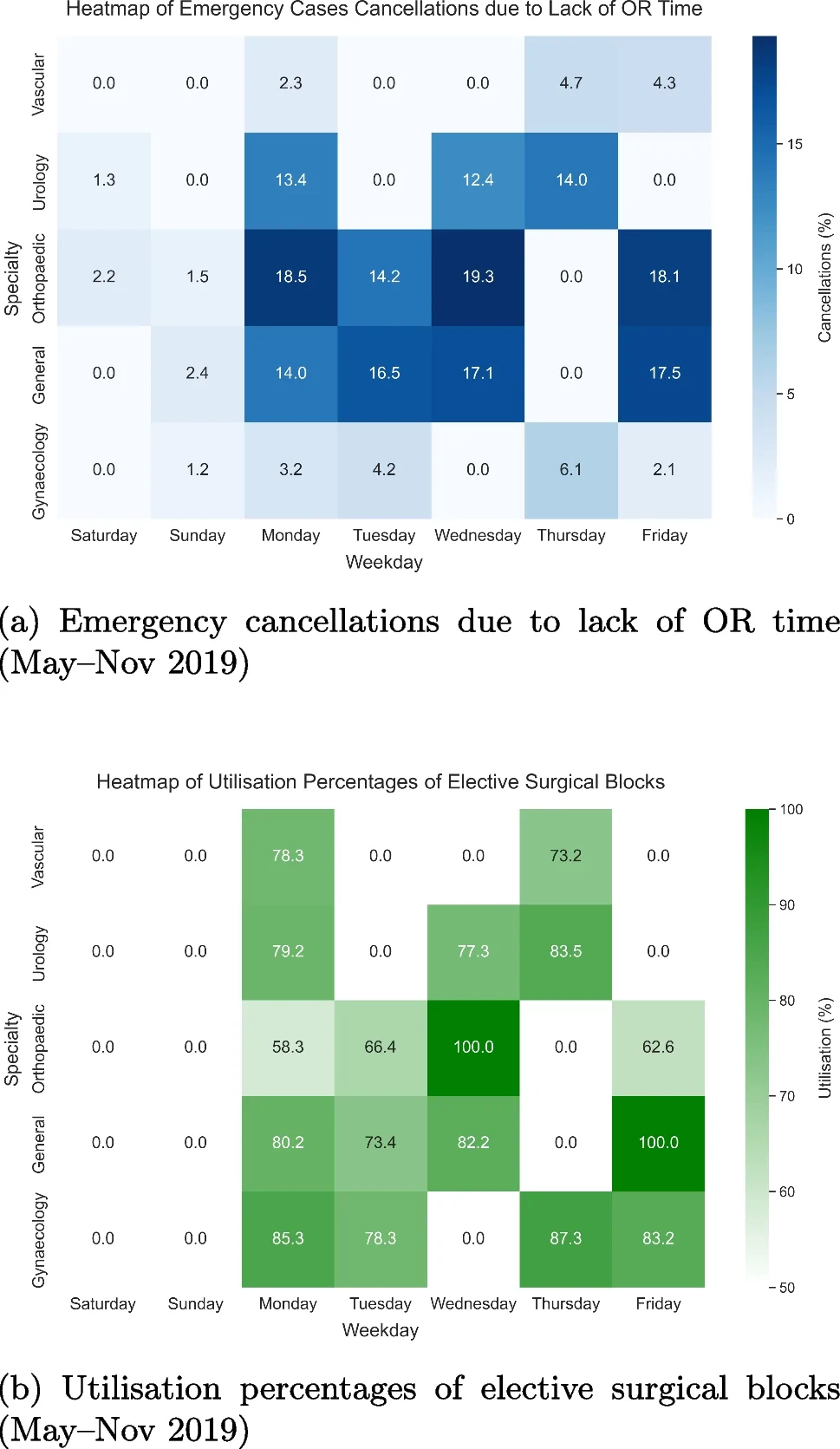
Emergency cancellations and utilisation percentages for several specialties (May–Nov 2019)

Our data analysis revealed two major inefficiencies:

- **High emergency case cancellations**: Many emergency cases were canceled due to insufficient OR availability. Figure 2a highlights the volume of emergency cancellations across multiple specialties, notably high during weekdays when emergency arrivals are more unpredictable. A cancellation rate of zero occasionally indicates that a specialty had no surgeries scheduled during that window, as indicated by the master surgery schedule in Fig. 1.
- **Low utilisation of elective blocks**: Elective OR blocks on the same weekdays had relatively low utilisation rates. Figure 2b reflects low utilisation rates of elective blocks, showing that some assigned times remain unused despite the growing demand for emergency cases of the same specialty.

**Fig. 3:**
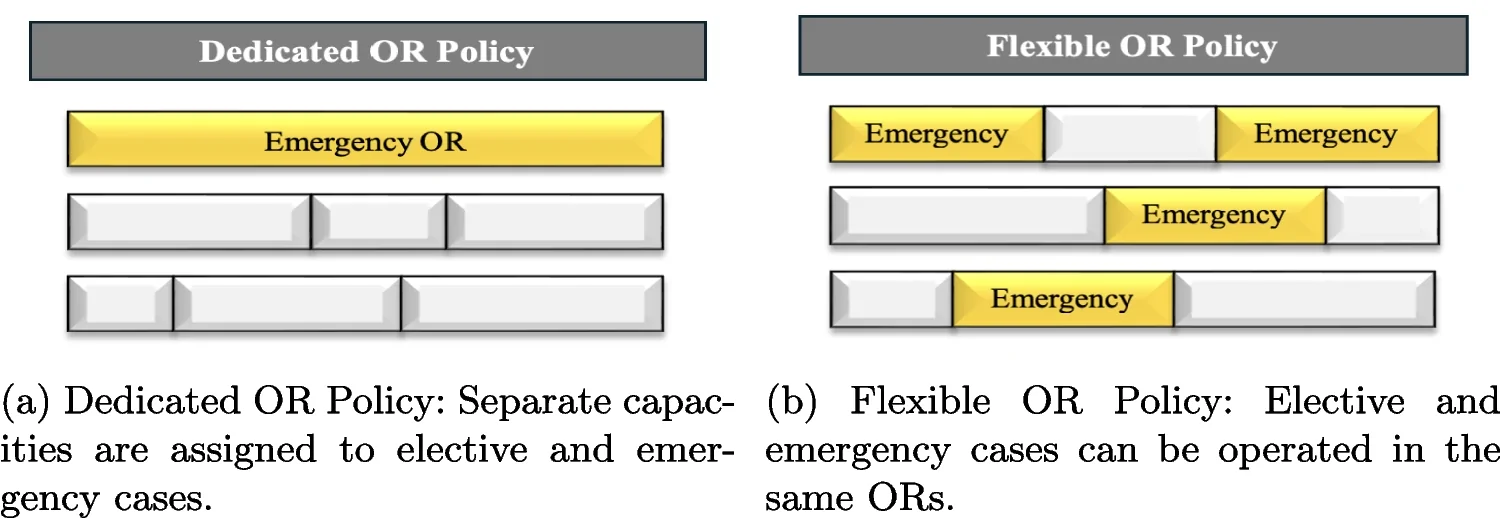
Comparison of (**a**) Dedicated and (**b**) Flexible OR Policies

The hospital currently follows a dedicated OR allocation (Fig. 3a), where elective and emergency cases are assigned to separate operating rooms. This rigid separation often leads to inefficiencies.

To overcome this limitation, we propose using **flexible ORs** instead of dedicated ORs. This means that both elective and emergency surgeries can be conducted in the same OR block, enhancing resource utilisation and reducing emergency cancellations (Fig. 3b).

Managing flexible ORs requires an effective scheduling strategy. Many hospitals use the BIM policy, inserting emergency cases into gaps within the elective schedule. However, BIM can cause problems when elective surgeries take longer than expected, causing cascading delays.

To address these limitations, we propose using buffer times—endogenous intervals between surgeries that account for the variability inherent in surgical procedures. Buffer times provide:

- A structured approach to integrating emergency cases without disrupting elective schedules.
- A safeguard against unpredictable delays, enhancing OR efficiency.

**Fig. 4:**
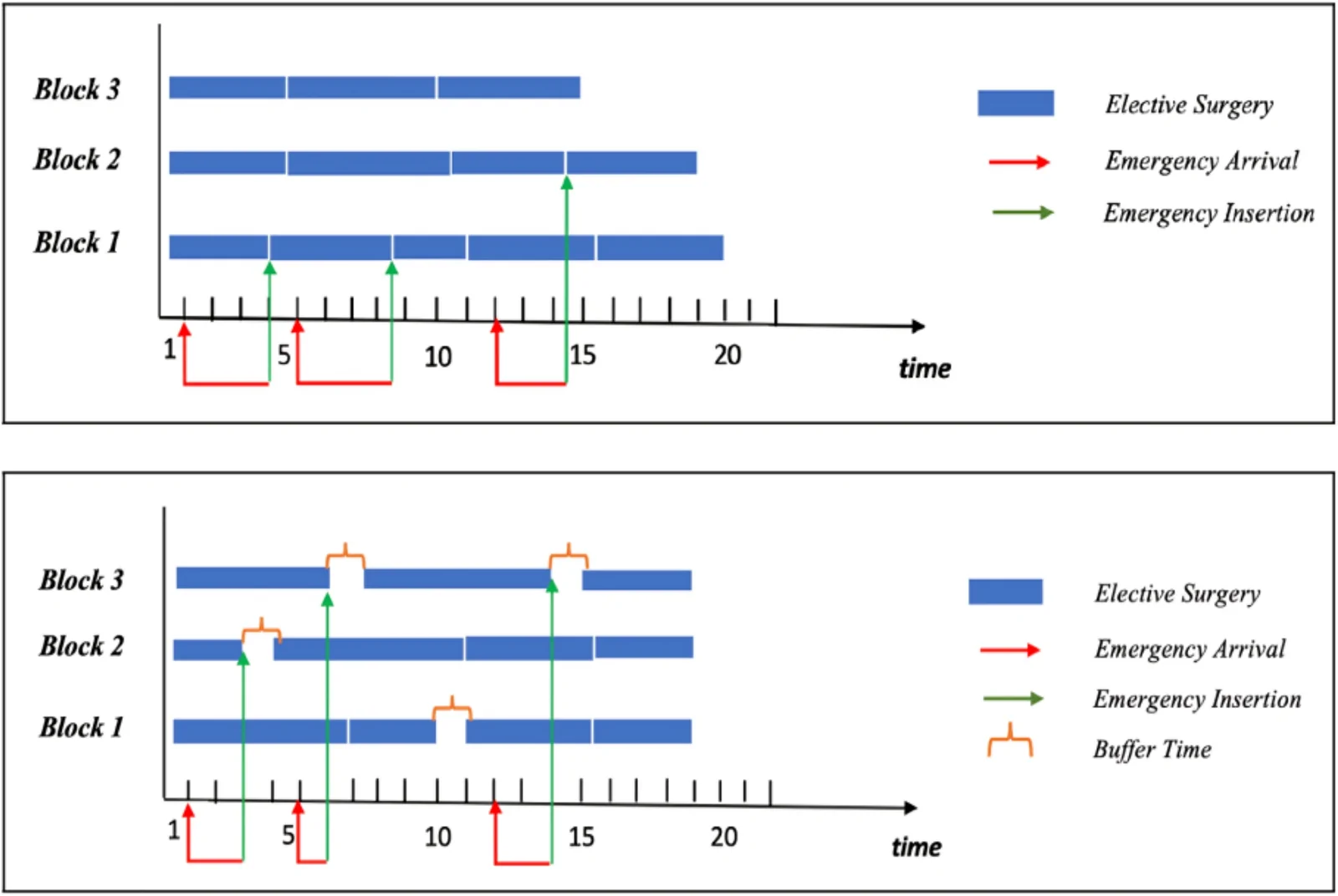
Illustrations of OR schedules with and without buffer times for emergency case arrivals

Figure 4 demonstrates the benefits of buffer times over BIM. By forecasting emergency demand and strategically placing buffer times, hospitals can improve flexibility and reduce cancellations while enhancing utilisation.

Building on these observations, our study develops a two-stage stochastic model to integrate flexible OR scheduling with optimally placed buffer times in advance. The first stage predicts emergency demand and determines buffer placements at the begining of the week. The second stage dynamically updates OR allocations based on real-time emergency arrivals and surgery durations. This approach improves OR efficiency by minimising emergency cancellations and reducing elective block underutilisation.

The following sections present our modeling framework and demonstrate its effectiveness in optimising hospital OR schedules.

## Literature review

OR scheduling involves complex decision-making to efficiently allocate limited resources, including ORs, staff, and recovery beds, while managing uncertainties such as unpredictable emergency arrivals, varying surgery durations, and fluctuating patient demand. Research has developed various operations research methodologies to address these challenges at different decision levels, including strategic, tactical, and operational planning (Koppka et al. [5]). The strategic level involves long-term allocation of surgical resources. The tactical level, typically spanning one to three months, involves creating an master surgery schedule. The operational level focuses on the specific scheduling of surgeries, including scheduling of dates for each surgery and the intra-day sequencing of elective cases (allocation scheduling). This study focuses on the operational level, where the scheduling of individual surgeries is most directly impacted by real-time uncertainties.

In the first step of operational-level optimisation, patients on the waiting list are assigned to specific ORs and surgery dates, then the order of each day’s operations and the precise time at which each procedure will be determined.

Kroer et al. [6] addressed a policy for optimising the daily allocation and order of elective procedures in a big hospital, considering the random arrivals of emergency patients and the unpredictability of surgical durations. They use clinical standards as constraints to determine the optimal trade-off between minimising the need for extra staff and making the most of the operating room’s available capacity.

In addition, Latorre et al. [7] presented a constrained optimisation method that integrates post-anesthesia bed availability, resource availability, and material availability into a single allocation scheduling problem considering the exact time of procedures. More recently, several other researchers have explored operational scheduling, focusing on both allocation and order of surgeries (see Saadouli et al. [8], Cardoen, Demeulemeester, and Beliën [9], and Xiao et al. [10]).

A major challenge in OR scheduling arises from uncertainties. For example, variability in surgery durations affects scheduling efficiency, often leading to idle time or unplanned overtime. Several studies, including those by Strum et al. [11], Min and Yih [12], Alvarez, Bowry, and Carter [13], and Van Riet and Demeulemeester [14], modeled surgery durations using a lognormal distribution, which closely fits empirical hospital data. This variability is influenced by patient-specific factors such as age, comorbidities, and surgical complexity, as explored by Olivares et al. [15] and Riise et al. [16].

Emergency surgeries further complicate scheduling, as their arrival times are difficult to predict. Some models account for emergency arrivals by reducing elective capacity to maintain available OR time, as seen in Min and Yih [12] and Rachuba and Werners [17]. Other studies explicitly modeled emergency arrivals as a Poisson process, capturing their stochastic nature, as demonstrated by Jebali and Diabat [18] and Truong [19].

Resource constraints further limit scheduling flexibility, particularly in hospitals with high surgical demand. ORs are the primary constraint, but other factors such as PACU and SICU bed availability, as well as staffing levels, also played a crucial role. Jonnalagadda et al. [20] reported that 15% of surgery cancellations resulted from a lack of PACU beds, while Utzolino et al. [21] found that premature SICU discharges due to bed shortages significantly increased readmission rates. Some researchers imposed strict OR capacity constraints to prevent excessive resource utilisation, as seen in Molina-Pariente et al. [22] and Roshanaei et al. [23]. Others allowed for limited overcapacity by incorporating overtime costs, as applied by Fei, Chu, and Meskens [24] and Wang, Tang, and Fung [25]. More adaptive models, such as those proposed by Neyshabouri and Berg [4], permitted short-term capacity violations but imposed penalties to balance efficiency and resource availability.

Several mathematical programming approaches have been applied to OR scheduling. Deterministic models assume all scheduling parameters are known in advance, simplifying computation but limiting adaptability to real-world uncertainty. Fei, Chu, and Meskens [26] formulated a binary integer programming model to optimise elective scheduling, while Molina-Pariente et al. [22] developed a similar framework incorporating heuristics to improve computational efficiency. Roshanaei et al. [23] extended this approach to multi-hospital networks using mixed-integer nonlinear programming. In contrast, stochastic programming explicitly models uncertainties in scheduling parameters. Denton et al. [27] and Min and Yih [12] developed stochastic integer programming models that account for emergency arrivals and surgery durations, using sample average approximation (SAA) to generate scenario-based solutions. Zhang et al. [28] further enhanced this approach with a hill-climbing algorithm to improve solution quality. Robust optimisation, as applied by Addis et al. [29], Denton et al. [27], and Neyshabouri and Berg [4], focuses on worst-case scenarios by optimising schedules against the most unfavorable conditions. In particular, Neyshabouri and Berg [4] proposed a two-stage robust model that explicitly accounts for uncertainty in both surgery duration and postoperative length-of-stay, with the objective of reducing the risk of downstream capacity shortfalls (for example, in PACU/ICU). While robust formulations provide strong protection under adverse realisations, they can be overly conservative in routine conditions, potentially reducing elective throughput compared with approaches that balance robustness against expected performance.

Managing OR capacity for emergency cases requires balancing elective surgery schedules while ensuring timely responses to unexpected emergencies. Hospitals typically adopt either a dedicated capacity or a flexible capacity policy. Dedicated capacity assigns specific ORs exclusively for emergency cases, allowing elective surgeries to proceed without disruption, but it risks prolonged wait times for emergencies when designated ORs reach full capacity (Denton et al. [27]). Flexible capacity, on the other hand, enables emergency cases to use any available OR, improving emergency responsiveness at the expense of greater scheduling uncertainty (Rachuba and Werners [17]). Comparative studies indicate that while the dedicated policy minimises elective case cancellations, the flexible policy provides a better balance between elective and emergency cases, necessitating sophisticated scheduling strategies.

Within flexible OR management, preventive scheduling approaches is one of the policies to mitigate disruptions. Preventive policies create an initial schedule that anticipates emergency cases by reserving OR time based on estimated demand. Lamiri et al. [30] and Tang and Wang [31] employed stochastic and robust optimisation techniques for this purpose, while Van Essen et al. [32] and Vandenberghe et al. [33] developed the Break-in-Moment (BIM) approach for inserting emergency surgeries between elective surgeries. However, BIMs can be ineffective when elective surgeries are lengthy, leaving fewer insertion opportunities. More recent studies by Aissaoui, Khlif, and Zeghal [34] and Xiao and Yoogalingam [35] propose buffer times to accommodate emergencies, offering greater flexibility than BIMs. Despite these innovations, most preventive scheduling models rely on deterministic or simplified representations of uncertainty, limiting their ability to adapt when real-time conditions deviate significantly from initial forecasts. Consequently, while these approaches improve OR adaptability, they often lack mechanisms for optimising real-time scheduling decisions, underscoring the need for integrated predictive and adaptive frameworks that better accommodate uncertainty and reduce cancellations.

Table 1 summarises selected surgery scheduling studies according to patient groups, sources of uncertainty, resources, emergency handling approach, and OR methodology. The emergency handling column distinguishes whether emergency cases are managed through BIM, policy-based buffers, or endogenous buffer placements.

**Table 1:** Overview of selected surgery scheduling studies referenced in the text

Reference	Patient Groups	Uncertainties	Resources	Emergency Handling	OR Methodologies
Kroer et al. [6]	Elective	Emergency arrivals, surgery durations	ORs, staff	BIM	Mathematical model balancing staff overtime and OR capacity
Latorre et al. [7]	Elective	Surgery durations	ORs, post-anesthesia beds, materials	None	Constrained optimisation for allocation scheduling
Saadouli et al. [8]	Elective	None	ORs	None	Operational-level scheduling approach
Cardoen et al. [9]	Elective	None	ORs	None	Operational planning and sequencing of surgeries
Xiao et al. [10]	Elective	Surgery durations	ORs	None	Combined allocation and sequencing; operational-level approach
Aissaoui, Khlif, and Zeghal [34]	Elective, emergency	Surgery durations	ORs	policy-based buffers	Preventive scheduling using buffer times
Xiao and Yoogalingam [35]	Elective, emergency	Surgery durations	ORs	policy-based buffers	Simulation optimisation; reserved-capacity policy
Fei, Chu, and Meskens [24]	Elective	None	ORs	None	Integer programming; column-generation-based heuristic
Denton et al. [27]	Elective	Surgery durations	ORs	None	Stochastic and robust optimisation; heuristics; integer L-shaped
Min and Yih [12]	Elective, emergency	Surgery durations, LOS in SICU, emergency arrivals	ORs, SICU	Policy-based capacity allocation	Stochastic programming; SAA
Wang, Tang and Fung [25]	Elective, emergency	Surgery durations, emergency arrivals	ORs	BIM	Chance-constrained optimisation; CGBH
Jebali and Diabat [18]	Elective	Surgery durations, LOS in SICU/ward	ORs, SICU, wards	None	Stochastic programming; SAA
Molina-Pariente et al. [22]	Elective	None	ORs, surgeons	None	Integer linear programming; heuristics
Addis et al. [29]	Elective	Surgery durations, patient arrivals	ORs	None	Robust optimisation
Neyshabouri and Berg [4]	Elective	Surgery durations, LOS	ORs, SICU	None	Robust optimisation; column-and-constraint generation
Rachuba and Werners [17]	Elective, emergency	Surgery durations, emergency arrivals	ORs	BIM	Robust multi-criteria optimisation; fuzzy sets
Roshanaei et al. [23]	Elective	None	ORs, surgical suites (multi-hospital)	None	Integer programming; logic-based Benders’ decomposition (LBBD)
Zhang et al. [28]	Elective	Surgery durations	ORs	None	Stochastic programming; SAA; exact hill-climbing algorithm
This study	Elective, emergency	Surgery durations, emergency arrivals	ORs	**Endogenous buffer placement**	Two-stage stochastic optimisation; SAA

Despite extensive research in OR scheduling, several key gaps remain. Much of the existing literature focuses predominantly on elective surgeries, often assuming a rigid separation between elective and emergency ORs. This overlooks operational realities in many hospitals, where ORs must be flexibly shared and real-time decisions are necessary. Furthermore, many models rely on static assumptions for emergency arrivals and solve the scheduling problem using deterministic formulations, failing to accommodate demand variability and uncertainty during schedule execution.

As shown in Table 1, previous studies have considered several approaches for handling emergency cases, including BIM strategies, and policy-based buffer strategies. While simple to implement, BIM becomes ineffective when elective surgeries overrun, reducing opportunities to accommodate emergency cases. Although buffer times have been explored as an alternative, most existing approaches use policy-based buffer durations based on deterministic assumptions, rather than adapting to stochastic variations in surgery durations and emergency arrivals. Moreover, buffer times are often treated in isolation rather than integrated into a unified, proactive scheduling framework. In contrast, the present study determines buffer timing and placement endogenously as part of the optimisation process, allowing buffer decisions to be informed by uncertainty in emergency arrivals and surgery durations.

Another important gap is the limited validation of models using realistic data. Many studies either rely on stylised examples that do not reflect the complexities of hospital operations. In contrast, our model is tested using a synthetic dataset calibrated to real hospital data, preserving realistic patterns in arrival distributions, durations, and resource constraints. This enhances both the validity and practical relevance of our findings.

This study addresses the above limitations by proposing a two-stage stochastic model that proactively integrates buffer time placement with predictive emergency demand and dynamically updates the schedule in response to realised uncertainties. By embedding this strategy into a scalable and data-driven framework, our model offers a practical solution for improving OR efficiency in hospitals managing both elective and emergency surgeries under uncertainty.

## The two-stage stochastic model

In this section we present the two-stage stochastic optimisation model used to jointly schedule elective and emergency surgeries under uncertainty. The model operates on a weekly planning horizon and is solved using the Sample Average Approximation (SAA) approach described later in Section 5.

### Problem definition and notation

We consider a public hospital where a set of elective patients $\mathcal {I} = \{1,2,\dots ,I\}$ must be scheduled during a one–week horizon together with a stochastic stream of emergency patients, where $I = |\mathcal {I}|$ is the total number of elective patients. Surgical capacity is organised into a finite set of surgical blocks $\mathcal {B} = \{1,2,\dots ,B\}$, where $B = |\mathcal {B}|$ is the number of blocks. Each block $b \in \mathcal {B}$ represents a combination of one operating room, one weekday, and one specialty in the MSS. In addition, we define a dummy block set $\mathcal {B}' = \{b'\}$ used to represent elective surgeries that are postponed beyond the current planning horizon, with $\mathcal {B} \cap \mathcal {B}' = \emptyset$.

Time is discretised into slots. Let $\mathcal {T}_r = \{1,2,\dots ,T_r\}$ denote the set of regular-time slots within a day (excluding overtime), where $T_r = |\mathcal {T}_r|$ is the number of regular slots, and $\mathcal {T} = \{1,2,\dots ,T\}$ the set of all available time slots, including both regular time and a maximum overtime period, where $T = |\mathcal {T}| \ge T_r$. We also introduce a dummy time set $\mathcal {T}' = \{t'\}$ for postponed surgeries, with $\mathcal {T} \cap \mathcal {T}' = \emptyset$.

Uncertainty is represented through a finite scenario set $\Phi = \{1,2,\dots ,K\}$, where K=|Φ| is the number of scenarios. For each scenario $\phi \in \Phi$ we let $\mathcal {J}_\phi = \{1,2,\dots ,J_\phi \}$ denote the corresponding set of emergency patients, with $J_\phi = |\mathcal {J}_\phi |$ the number of emergencies in scenario ϕ. The random arrival time of emergency patient $j \in \mathcal {J}_\phi$ in scenario ϕ is denoted by $E_j^\phi$, and the random surgery durations of elective and emergency patients are denoted by $L_i^\phi$ and $L_j^\phi$, respectively. For each scenario ϕ, we denote by P(ϕ) the probability of occurrence of that scenario.

Each surgical block $b \in \mathcal {B}$ has a regular open duration R and a maximum allowable overtime O. Since blocks belonging to the same specialty share identical regular and overtime durations, we omit block-specific subscripts for R and O. Emergency patients are subject to a maximum waiting time $W_{\max }$; an emergency arriving at time $E_j^\phi$ must start surgery no later than $E_j^\phi + W_{\max }$ in scenario ϕ.

The model accounts for several cost components. For each elective patient $i \in \mathcal {I}$ and block $b \in \mathcal {B}$, we denote by $C^a_{ib}$ the allocation cost of performing surgery i in block b. If elective surgery i is postponed to the dummy block $b' \in \mathcal {B}'$, a postponement cost $C^a_{ib'}$ is incurred. Cancelling an elective surgery i yields a penalty $C^c_i$. For each emergency patient $j \in \mathcal {J}_\phi$, we denote by $C^a_j$ the cost of allocating patient j to a block and by $C^c_j$ the penalty for cancelling this emergency case. The overtime cost of block $b \in \mathcal {B}$ is given by $C^o_b$ per unit of overtime.

Scheduling decisions are captured by first-stage and second-stage variables. In the first stage, for each elective patient $i \in \mathcal {I}$, block $b \in \mathcal {B}$ and regular time slot $t \in \mathcal {T}_r$, we define a binary variable $x_{ibt}$ which takes value 1 if surgery i is planned to begin at time t in block b, and 0 otherwise. If elective surgery i is planned to be postponed to the next planning horizon, we use the binary variable $x_{ib't'}$ to indicate that it is assigned to dummy block $b' \in \mathcal {B}'$ at dummy time $t' \in \mathcal {T}'$. Preventive buffer times are represented by binary variables $h_{bt}$, equal to 1 if time slot $t \in \mathcal {T}_r$ in block $b \in \mathcal {B}$ is reserved as buffer, and 0 otherwise. Importantly, $h_{bt}$ is an endogenous decision variable rather than a predefined parameter. Therefore, the model determines which time slots should be reserved as buffers within each surgical block, allowing buffer placement to differ across blocks, weekdays, and specialties.

Second-stage (recourse) decisions are scenario dependent. For each scenario $\phi \in \Phi$, elective case $i \in \mathcal {I}$, block $b \in \mathcal {B}$, and time slot $t \in \mathcal {T}$, we define a binary variable $y^\phi _{ibt}$ which equals 1 if surgery i actually starts at time t in block b under scenario ϕ. Binary variables $y^{+\phi }_{ibt}$ indicate whether elective case i is occupying block b at time t in scenario ϕ, and $y^{-\phi }_i$ indicates cancellation of elective surgery i under scenario ϕ.

Similarly, for each emergency patient $j \in \mathcal {J}_\phi$, block $b \in \mathcal {B}$ and time $t \in \mathcal {T}$, binary variables $z^\phi _{jbt}$ and $z^{+\phi }_{jbt}$ represent, respectively, the start time and occupancy of emergency case j in block b under scenario ϕ. We also use a binary variable $z^{-\phi }_j$ to indicate cancellation of emergency case j. Finally, continuous non-negative variables $o_b^\phi$ measure the overtime used in block b in scenario ϕ.

All sets, decision variables, and parameters are summarised in Table 2.

**Table 2:** Notation

Sets	
$\mathcal {I}$	Set of elective patients, $\mathcal {I}=\{1,2,\dots ,I\}$
$\mathcal {B}$	Set of surgical blocks, $\mathcal {B}=\{1,2,\dots ,B\}$
$\mathcal {B}'$	Set of dummy block for postponed surgeries, $\mathcal {B}' = \{b'\}$,
	where $\mathcal {B} \cap \mathcal {B}' = \emptyset$
Φ	Set of scenarios, $\Phi =\{1,2,\dots ,K\}$
$\mathcal {J}_\phi$	Set of emergency patients in scenario ϕ, $\mathcal {J}_\phi =\{1,2,\dots ,J_\phi \}$
$\mathcal {T}_r$	Set of regular time slots in a day (excluding overtime slots),
	$$\mathcal {T}_r=\{1,2,\dots ,T_r\}$$
$\mathcal {T}$	Set of total available time slots in a day, including both
	regular and maximum overtime slots, $\mathcal {T}=\{1,2,\dots ,T\}$
$\mathcal {T}'$	Set of dummy time slots for postponed surgeries, $\mathcal {T}' = \{t'\}$
	where $\mathcal {T} \cap \mathcal {T}' = \emptyset$
$\mathcal {U}$	Bounded (budgeted) uncertainty set defining all feasible
	realisations of ξ in the robust optimisation model
Decision Variables	
$x_{ibt}$	1 if the elective case $i \in \mathcal {I}$ is planned to begin surgery at time
	$t \in \mathcal {T}_r$ in block $b \in \mathcal {B}$, 0 otherwise
$x_{ib't'}$	1 if the elective case $i \in \mathcal {I}$ is planned to be postponed into a
	dummy block $b' \in \mathcal {B}'$ at a dummy time $t' \in \mathcal {T}'$, 0 otherwise
$h_{bt}$	1 if block $b \in \mathcal {B}$ at time $t \in \mathcal {T}_r$ is designated as buffer time by the optimisation model, 0 otherwise
$y^\phi _{ibt}$	1 if the elective case $i \in \mathcal {I}$ actually starts surgery in block $b \in \mathcal {B}$
	at time $t \in \mathcal {T}$ under scenario $\phi \in \Phi$, 0 otherwise
$y^{+\phi }_{ibt}$	1 if the elective case $i \in \mathcal {I}$ is using block $b \in \mathcal {B}$
	at time $t \in \mathcal {T}$ under scenario $\phi \in \Phi$, 0 otherwise
$y^{-\phi }_{i}$	1 if the elective case $i \in \mathcal {I}$ is canceled under scenario $\phi \in \Phi$,
	0 otherwise
$z^\phi _{jbt}$	1 if the emergency case $j \in \mathcal {J}_\phi$ begins surgery at time *t* in block $b \in \mathcal {B}$
	under scenario $\phi \in \Phi$, 0 otherwise. This variable is only defined for time slots
	*t* satisfying $E^\phi _j \le t \le E^\phi _j + W_{\max }$.
$z^{+\phi }_{jbt}$	1 if the emergency case $j \in \mathcal {J}_\phi$ is using block $b \in \mathcal {B}$
	at time $t \in \mathcal {T}$ under scenario $\phi \in \Phi$, 0 otherwise
$z^{-\phi }_{j}$	1 if the emergency case $j \in \mathcal {J}_\phi$ is canceled under scenario $\phi \in \Phi$,
	0 otherwise
$o^\phi _{b}$	Overtime of surgical block *b* in scenario ϕ
Parameters	
$E^\phi _j$	Arrival time of emergency case $j \in \mathcal {J}_\phi$ in scenario $\phi \in \Phi$
P(ϕ)	Probability of occurrence of scenario $\phi \in \Phi$
$C^a_{ib}$	Cost of allocating elective patient $i \in \mathcal {I}$ to block $b \in \mathcal {B}$
$C^a_{ib'}$	Cost of postponing surgery $i \in \mathcal {I}$ to a dummy block $b' \in \mathcal {B}'$
$C^c_{i}$	Cost of cancelling elective case $i \in \mathcal {I}$
$C^a_{j}$	Cost of allocating emergency patient $j \in \mathcal {J}_\phi$ to block $b \in \mathcal {B}$
$C^c_{j}$	Cost of cancelling emergency case $j \in \mathcal {J}_\phi$
$C^o_{b}$	Cost of overtime usage of block $b \in \mathcal {B}$
$L_{\min }$	Minimum duration of each surgery
$L_i^\phi$	Actual surgery duration of elective case $i \in \mathcal {I}$
	in scenario $\phi \in \Phi$
$L_j^\phi$	Actual surgery duration of emergency case
	$j \in \mathcal {J}_\phi$ in scenario $\phi \in \Phi$
*R*	Regular open duration of each surgical block $b \in \mathcal {B}$
*O*	Maximum allowable overtime of each surgical block $b \in \mathcal {B}$
$W_{\max }$	Maximum waiting time that an emergency case can wait
ξ	Uncertain parameters, including emergency arrivals and
	surgery durations; common to stochastic and robust models
$\bar{\xi }$	Nominal (mean) values of the uncertain parameters
	(Poisson and lognormal means)
δ	Maximum allowable deviations for uncertain parameters
η	Normalized deviation multiplier controlling the direction
	and magnitude of perturbations, $\eta \in [-1,1]$
Γ	Uncertainty budget controlling how many parameters can
	simultaneously take their worst-case deviations

### Modelling assumptions

The model is based on the following assumptions, which formalise the operational setting described above:

- **A1**. The full set of elective patients $\mathcal {I}$ is known at the beginning of the planning horizon. Each patient $i \in \mathcal {I}$ can undergo surgery in at most one block during the week; that is, an elective case cannot be split across blocks or allocated more than once.
- **A2**. For each scenario $\phi \in \Phi$, the number of emergency patients $|\mathcal {J}_\phi |$ is a Poisson-distributed random variable. For every emergency case $j \in \mathcal {J}_\phi$, the corresponding arrival time $E_j^\phi$ is exponentially distributed. This representation captures the random and memoryless nature of emergency arrivals and aligns with established approaches in the literature (see Jebali and Diabat [18]; Truong [19]).
- **A3**. Surgery durations for elective and emergency patients, $L_i^\phi$ and $L_j^\phi$, are treated as random variables following lognormal distributions fitted to synthetic data. This choice reflects the empirically observed skewness and variability in surgical times and aligns with the most common practice in the literature (see Strum et al. [11], Min and Yih [12], and Van Riet and Demeulemeester [14]).
- **A4**. For modeling simplicity and computational tractability, we assume that supporting resources—such as surgical teams and equipment—are available as planned according to the schedule, within the hospital’s capacity limits. This abstraction allows us to concentrate on the core contributions of the model, namely the strategic placement of preventive buffer times and the stochastic balancing of elective and emergency cases. We acknowledge, however, that in practice shortages such as staff absences or equipment failures can cause cancellations or delays. Explicitly modeling these resource constraints would enhance realism and represents an important avenue for future research.
- **A5**. Overtime is allowed for each surgical block up to a capped limit (*O*), and the associated overtime costs ($C^o_{b}$) are included in the model’s objective function. The scheduling decisions consider overtime policies to balance the trade-off between extending operating hours and the costs incurred, aiming to minimise unnecessary overtime while ensuring all surgeries are accommodated.

### First-stage formulation

In the first stage, preliminary scheduling decisions are made using deterministic information. Elective surgeries are scheduled based on a conservative minimum duration $L_{\min }$ for each surgery. Any elective case that cannot be accommodated within the available regular-time capacity is assigned to the dummy block $b' \in \mathcal {B}'$ and dummy time $t' \in \mathcal {T}'$, and is interpreted as postponed to a future planning horizon. Time slots that are not used for elective cases may be designated as buffer time ($h_{bt}$) and can later be used to accommodate emergency arrivals or delays in realised durations. Thus, the amount and placement of buffer time are not fixed in advance or imposed by a policy rule; they emerge from the optimisation model through the interaction between first-stage scheduling decisions and expected second-stage recourse costs.

1 $$\begin{aligned} f_1= &   \min \Bigg ( \sum _{i \in \mathcal {I}} \sum _{b \in \mathcal {B}} \sum _{t \in \mathcal {T}_r} C^a_{ib} x_{ibt} + \sum _{i \in \mathcal {I}}\sum _{b' \in \mathcal {B}'}\sum _{t' \in \mathcal {T}'} C^a_{ib'} x_{ib't'} \nonumber \\  &   \qquad + \mathbb {E}(Q(\boldsymbol{x}, \phi )) \Bigg ) \end{aligned}$$

2 $$\begin{aligned} \text {s.t.}\quad&\sum _{b \in \mathcal {B}} \sum _{t \in \mathcal {T}_r} x_{ibt} + \sum _{b' \in \mathcal {B}'} \sum _{t' \in \mathcal {T}'} x_{ib't'} = 1, \quad \forall i \in \mathcal {I} \end{aligned}$$

3 $$\begin{aligned}&\sum _{i \in \mathcal {I}} \sum _{t \in \mathcal {T}_r} L_{\text {min}} x_{ibt} \le R, \quad \forall b \in \mathcal {B} \end{aligned}$$

4 $$\begin{aligned}&\sum _{i \in \mathcal {I}} x_{ibt} \le 1, \quad \forall b \in \mathcal {B}, \; t \in \mathcal {T}_r \end{aligned}$$

5 $$\begin{aligned}&{ h_{bt} + \sum _{i \in \mathcal {I}} \sum _{s : s + L_{\min } - 1 \ge t} x_{ibs} \le 1,} \qquad \forall b \in \mathcal {B}, \; t \in \mathcal {T}_r\end{aligned}$$

6 $$\begin{aligned}&x_{ibt} \in \{0,1\}, \quad \forall i \in \mathcal {I}, \; b \in \mathcal {B}, \; t \in \mathcal {T}_r \end{aligned}$$

7 $$\begin{aligned}&x_{ib't'} \in \{0,1\}, \quad \forall i \in \mathcal {I}, \; b' \in \mathcal {B}', \; t' \in \mathcal {T}' \end{aligned}$$

The objective function $$ f_1 $$ in Eq. 1 aims to minimise the total cost associated with scheduling elective surgeries. It consists of three main terms. The first term, $$ \sum _{i \in \mathcal {I}} \sum _{b \in \mathcal {B}} \sum _{t \in \mathcal {T}_r} C^a_{ib} x_{ibt} $$, represents the total cost of scheduling elective patient i in block b at time t during regular operating hours. The second term, $$ \sum _{i \in \mathcal {I}} \sum _{b' \in \mathcal {B}'} \sum _{t' \in \mathcal {T}'} C^a_{ib'} x_{ib't'} $$, accounts for the cost of postponing elective surgeries to a dummy block $b^{\prime }$ at a dummy time $t^{\prime }$, indicating that the surgery cannot be scheduled within the current planning horizon. These dummy variables are not part of the actual sets $$ \mathcal {B} $$ and $$ \mathcal {T} $$ and serve to represent postponed surgeries. The third term, $$ \mathbb {E}(Q(\boldsymbol{x}, \phi )) $$, represents the expected cost of second-stage decisions, incorporating uncertainties across all scenarios ϕ.

Constraint Eq. 2 ensures that each elective surgery i is assigned exactly once, either to a specific block b at time t during regular hours or to the dummy block $b^{\prime }$ at a dummy time $t^{\prime }$ if it is postponed. This constraint guarantees that no elective surgery is scheduled more than once or left unscheduled without being marked as postponed.

Constraint Eq. 3 ensures that the total minimum required time for all elective surgeries scheduled in block b does not exceed the regular open duration R of that block. The left-hand side sums the minimum durations $L_{\text {min}}$ for all surgeries assigned to block b, reflecting a conservative estimate to prevent overutilisation of operating room time.

Constraint Eq. 4 enforces that at most one elective surgery can start at any given time t in block b during regular hours. This prevents scheduling conflicts by ensuring that two surgeries are not assigned to start at the same time in the same operating room, thereby avoiding double-booking of resources.

Constraint Eq. 5 ensures that, at any given time slot *t* within block *b*, either buffer time $h_{bt}$ is scheduled or an elective surgery is occupying the block—but not both simultaneously. Here, $h_{bt}$ is a binary variable indicating whether buffer time is allocated in block *b* at time *t*. The summation term $\sum _{i \in \mathcal {I}} \sum _{s: s + L_{\min } - 1 \ge t} x_{ibs}$ captures all elective surgeries *i* that could be ongoing at time *t* in block *b*. Since each surgery has a minimum duration $L_{\min }$, a surgery starting at time *s* occupies the block from *s* to $s + L_{\min } - 1$, meaning that time *t* is occupied if $s \le t \le s + L_{\min } - 1$. This constraint therefore prevents overlaps and ensures that time slot *t* cannot be simultaneously assigned to both a buffer and a surgery. Unlike policy-based buffer approaches, Constraint Eq. 5 allows the optimisation model to determine which time slots should be reserved as buffer time within each surgical block.

Constraints Eqs. 6 and 7 specify the binary nature of the decision variables $x_{ibt}$ and $x_{ib't'}$. They can take the value of 1 if the elective surgery is scheduled to start at the corresponding block and time, or 0 otherwise. This binary restriction is essential for modeling the scheduling decisions accurately within the optimisation framework.

In summary, the first-stage model establishes an initial schedule for elective surgeries based on minimum surgery durations, without yet considering uncertainties. It allocates surgeries to available time slots while respecting operating room capacities and ensuring no scheduling conflicts occur. Buffer times are incorporated into the schedule to provide flexibility for unexpected events in the second stage. The expected cost term $\mathbb {E}(Q(x, \phi ))$ prepares the model to incorporate stochastic elements in the subsequent stage, where actual durations and emergency cases are considered to adjust the schedule accordingly.

### Interaction between the two stages

The first-stage decisions are not made in isolation; they are strategically influenced by the anticipated outcomes of the second stage. The expected cost term $\mathbb {E}(Q(\boldsymbol{x}, \phi ))$ in the objective function represents the feedback loop from the second stage to the first stage. By including this term, the model effectively anticipates the uncertainties related to actual surgery durations and emergency arrivals.

This means that the initial scheduling of elective surgeries ($x_{ibt}$) and allocation of buffer times ($h_{bt}$) are optimised not just based on immediate costs, but also by considering how these decisions will perform under various future scenarios. The model seeks to minimise total expected costs by balancing the trade-off between efficient scheduling and the flexibility required to accommodate uncertainties.

For instance, if the model anticipates a high likelihood of emergency arrivals during certain time slots, it may allocate more buffer time in those periods in the first stage. Similarly, surgeries that are more critical or have higher cancellation costs might be scheduled in a way that reduces the risk of being affected by uncertainties.

This integrated approach ensures that the first-stage schedule is robust and cost-effective, leading to improved resource utilisation and patient care.

### Stage two

In the second stage, those preliminary decisions of the first stage are adjusted based on real-time information and the uncertainties that arise during each day. The uncertain parameters in this model include the actual surgery durations and the emergency case arrivals, both of which are modeled stochastically based on the real-world-inspired data. Multiple scenarios are generated for each potential outcome, with variations in random parameters (e.g., log-normal for surgery durations and Poisson for emergency arrivals). The model then adjusts the initial schedule dynamically, either by reallocating the buffer times to emergency surgeries or by making changes to the overtime requirements or cancellations.

8 $$\begin{aligned} \mathbb {E}(Q(\boldsymbol{x},\phi )) \!= &   \! \sum _{\phi \in \Phi } P(\phi ) \Bigg ( \sum _{j \in \mathcal {J}_\phi } \sum _{b \in \mathcal {B}} \sum _{t \in \mathcal {T}} C^a_j z_{jbt}^\phi \!+\!\!\! \sum _{j \in \mathcal {J}_\phi } C^c_j z_j^{-\phi }\nonumber \\  &   + \sum _{i \in \mathcal {I}} C^c_i y_i^{-\phi } + \sum _{b \in \mathcal {B}} C^o_b o_b^\phi \Bigg ). \end{aligned}$$

In this objective function (Eq. 8), $\mathbb {E}(Q(\boldsymbol{x},\phi ))$ reflects the expected cost of decisions made in the second stage, across all possible scenarios ϕ, each of which accounts for uncertainties. The term P(ϕ) represents the probability of each scenario.

The objective function captures key aspects of surgery scheduling costs. The first term represents the cost of scheduling emergency surgeries across time slots and blocks, which is essential for managing the unpredictable nature of emergencies. The second term reflects the cost of canceling emergency surgeries when they cannot be completed within the allowable time, addressing both urgency and resource limitations. The third term accounts for the cost of canceling elective surgeries due to disruptions, and the final term covers overtime costs for surgeries that exceed regular hours, ensuring that the model considers both scheduling efficiency and operational expenses.

Overall, the objective function minimises the total expected cost by anticipating various uncertainties that arise during the scheduling process and optimising for both efficiency and flexibility in the second stage.

Before diving into the constraints of the second-stage model, it’s important to recognize that these constraints are scenario-specific, meaning they apply individually to each scenario ϕ. The second stage serves to refine the first-stage scheduling decisions by adjusting them based on the real-world outcomes.

These constraints are designed to ensure that surgeries do not overlap, that they are performed continuously within the available time, and that they do not exceed regular and overtime hours allocated to each surgical block. Additionally, the constraints ensure consistency between the scheduled and actual surgery start times, define the rules for cancellations or postponements, and manage buffer times efficiently. Together, they create a robust system capable of adapting to uncertainties while optimising the use of surgical resources.

- **Non-overlapping of surgeries**

9 $$\begin{aligned} \sum _{i \in \mathcal {I}} y^{+\phi }_{ibt}+\sum _{j \in \mathcal {J}_\phi } z^{+\phi }_{jbt}\le 1, \;\;\;\; \forall {b\in \mathcal {B}, t\in \mathcal {T}, \phi \in \Phi } \end{aligned}$$

Equation 9 ensures that at most one surgery (either elective or emergency) can use a surgical block *b* at any time *t* under each scenario ϕ. This is to ensure that the surgical block is not over-utilised.

- **Continuation of surgeries**

10 $$\begin{aligned}  &   \sum _{m=t}^{t+L^\phi _i-1} y^{+\phi }_{ibm} \ge L^\phi _i y^\phi _{ibt}, \nonumber \\  &   \forall i \in \mathcal {I}, \; b \in \mathcal {B}, \; \phi \in \Phi , \; t = 1, \dots , |\mathcal {T}| - L^\phi _i + 1 \end{aligned}$$

11 $$\begin{aligned}  &   \sum _{m=t}^{t+L^\phi _j-1} z^{+\phi }_{jbm} \ge L^\phi _j z^\phi _{jbt}, \nonumber \\  &   \forall \phi \in \Phi , \; j \in \mathcal {J}_\phi , \; b \in \mathcal {B}, \; t = 1, \dots , |\mathcal {T}| - L^\phi _j + 1 \end{aligned}$$

Equation 10 ensures that if an elective surgery *i* is scheduled to start at time *t* in block *b* under scenario ϕ, represented by $y^\phi _{ibt} = 1$, the block must be reserved for the full duration of the surgery, $L^\phi _i$. The left-hand side sums the usage of block *b* from time *t* to $t + L^\phi _i - 1$, ensuring continuous use of the block for the surgery’s entire length.

Equation 11 mirrors the same logic for emergency surgeries.

Basically, Eqs. 10 and 11 define the variables $\boldsymbol{y^{+\phi }}$ and $\boldsymbol{z^{+\phi }}$, ensuring the block is continuously utilised for the entire surgery duration.

- **Total duration of surgeries**

12 $$\begin{aligned} \sum _{i \in \mathcal {I}}\sum _{t \in \mathcal {T}} y^\phi _{ibt} L_i^{\phi } \!+\! \sum _{j \in \mathcal {J^{\phi }}}\sum _{t\in \mathcal {T}} z^\phi _{jbt} L_j^{\phi } \le R+O, \;\; \forall b \in \mathcal {B}, \phi \!\in \! \Phi \end{aligned}$$

Equation 12 ensures that the total duration of surgeries (including both elective and emergency surgeries) in each scenario ϕ in each block *b* should be less than or equal to the total available time in that block, including both regular time (*R*) and overtime (*O*).

- **Consistency of scheduled and actual start times**

13 $$\begin{aligned} \sum _{t \in \mathcal {T}_r} t x_{ibt} \le \sum _{t \in \mathcal {T}} t y^{\phi }_{ibt}, \quad \forall i \in \mathcal {I}, b \in \mathcal {B}, \phi \in \Phi \end{aligned}$$

Equation 13 ensures that the actual start time of a surgery in any given scenario ($y^{\phi }_{ibt}$) is not earlier than its scheduled start time ($x_{ibt}$). It helps maintain consistency between the scheduling plan and the actual execution, especially in a stochastic setting where multiple scenarios are considered.

- **Calculating overtime**

Overtime equals the number of occupied slots strictly between $\mathcal {T}_r$ and $\mathcal {T}$ and is denoted by $o^\phi _{b}$:

14 $$\begin{aligned} \sum _{m \in \mathcal {T} \setminus \mathcal {T}_r} \left( \sum _{i \in \mathcal {I}} y^{+\phi }_{ibm} + \sum _{j \in \mathcal {J}\phi } z^{+\phi }_{jbm} \right) \le o^\phi _{b}, \quad \forall b \in \mathcal {B}, \phi \in \Phi . \end{aligned}$$

Equation 14 defines the overtime $o^\phi _{b}$ for each surgical block *b* in scenario ϕ. The regular operating window is represented by $\mathcal {T}_r$, while $\mathcal {T}$ denotes the full scheduling horizon. The term $\mathcal {T} \setminus \mathcal {T}_r$ therefore captures all time slots that lie beyond regular hours. The inner summations count the number of slots occupied by elective ($y^{+\phi }_{ibm}$) and emergency ($z^{+\phi }_{jbm}$) surgeries during those overtime slots. This value is bounded by the overtime variable $o^\phi _{b}$, which measures how many time slots extend beyond the planned working hours.

- **Elective case cancellations**

This section defines the conditions under which a scheduled elective surgery is considered canceled. The cancellation rules are as follows:

$$\begin{aligned} \text {If }&\sum _{t \in \mathcal {T}_r}\sum _{b \in \mathcal {B}} x_{ibt} = 1 \text { and } \sum _{t \in \mathcal {T}}\sum _{b \in \mathcal {B}} y^\phi _{ibt} = 0, \\&\text {then } y^{-\phi }_{i} = 1, \text { meaning the surgery was scheduled but not performed.} \\ \text {If }&\sum _{t \in \mathcal {T}_r}\sum _{b \in \mathcal {B}} x_{ibt} = 0 \text { and } \sum _{t \in \mathcal {T}}\sum _{b \in \mathcal {B}} y^\phi _{ibt} = 0, \\&\text {then } y^{-\phi }_{i} \!=\! 0, \text { meaning the surgery was neither scheduled nor performed.} \\ \text {If }&\sum _{t \in \mathcal {T}_r}\sum _{b \in \mathcal {B}} x_{ibt} = 1 \text { and } \sum _{t \in \mathcal {T}}\sum _{b \in \mathcal {B}} y^\phi _{ibt} = 1, \\&\text {then } y^{-\phi }_{i} = 0, \text { meaning the surgery was scheduled and performed.} \end{aligned}$$

So, based on these conditions, we have:

15 $$\begin{aligned} y^{-\phi }_{i} = \sum _{t \in \mathcal {T}_r}\sum _{b \in \mathcal {B}} x_{ibt} - \sum _{t \in \mathcal {T}}\sum _{b \in \mathcal {B}} y^\phi _{ibt}, \quad \forall i \in \mathcal {I},\; \phi \in \Phi . \end{aligned}$$

This constraint (Eq. 15) ensures that $y^{-\phi }_{i}$ is set to 1 only when a surgery is scheduled but not performed, and 0 in all other cases. Additionally, the condition $y^{-\phi }_{i} \in \{0, 1\}$ prevents the case where $\sum _{t \in \mathcal {T}_r}\sum _{b \in \mathcal {B}} x_{ibt} = 0$ and $\sum _{t \in \mathcal {T}}\sum _{b \in \mathcal {B}} y^\phi _{ibt} = 1$, ensuring that a surgery cannot be performed unless it was scheduled in advance.

- **Emergency case cancellations**

16 $$\begin{aligned} \sum _{b \in \mathcal {B}} \sum _{t \in \mathcal {T}} z^\phi _{jbt} + \sum _{b \in \mathcal {B}} z^{-\phi }_{j} = 1, \quad \forall j \in \mathcal {J}_\phi , \forall \phi \in \Phi \end{aligned}$$

This constraint enforces that each emergency surgery j is assigned exactly one outcome: it is either scheduled once in a block $b \in \mathcal {B}$ at a time $t \in \mathcal {T}$, or it is canceled. In other words, the model cannot simultaneously schedule and cancel the same surgery, thereby ensuring consistency in the decision-making process.

Note that $z^\phi _{jbt}$ is only defined for time slots t that satisfy the condition $E^\phi _j \le t \le E^\phi _j + W_\text {max}$.

This feature is critical for the model, as it guarantees proper handling of emergency cases and prevents logical inconsistencies in the scheduling framework.

- **Non-overlapping of elective surgeries and buffer times**

17 $$\begin{aligned}  &   y^\phi _{ibt} + h_{bm} \;\le 1, \qquad \forall i \in \mathcal {I},\; b \in \mathcal {B},\; \phi \in \Phi ,\; t \in \mathcal {T},\nonumber \\  &   m = t,\dots ,t+L_i^\phi -1 \end{aligned}$$

Equation 17 ensures that if an elective surgery *i* starts at time *t* in block *b* ($y^\phi _{ibt} = 1$), then for every slot *m* within its planned duration $[t, t + L_i^\phi - 1]$ no buffer time $h_{bm}$ may exist. This prevents any overlap between elective surgeries and buffer periods that are reserved for emergency cases. Consequently, no elective surgery can start during or so close to a reserved buffer period that its duration would overlap with the buffer.

- **Binary variables**

18 $$\begin{aligned} z^\phi _{jbt}, z^{+\phi }_{jbt} , z^{-\phi }_{j} \in \{0,1\}, \;\;\;\; \forall {j\in \mathcal {J}, b\in \mathcal {B}, t\in \mathcal {T}, \phi \in \Phi } \end{aligned}$$

19 $$\begin{aligned} y^\phi _{ibt}, y^{+\phi }_{ibt}, y^{-\phi }_{i} \in \{0,1\}, \;\;\;\; \forall {i \in \mathcal {I}, b \in \mathcal {B}, t\in \mathcal {T}, \phi \in \Phi } \end{aligned}$$

Equations 18 and 19 define $z^\phi _{jbt}$, $z^{+\phi }_{jbt}$, $z^{-\phi }_{j}$, $y^\phi _{ibt}$ and $y^{+\phi }_{ibt}$ as binary decision variables.

## Solution approach

This section outlines the solution methodology—SAA—used to solve the two-stage stochastic model introduced in Section 4. In addition to the two-stage stochastic model solved by SAA, we use several comparative benchmark solutions to evaluate the efficiency and performance improvements of our approach. Specifically, four alternative benchmark solutions are considered to represent distinct decision-making strategies under varying levels of uncertainty (Section 5.1).

Following the presentation of these benchmark solutions, we describe the computational tools and solvers used for implementation and performance analysis (Section 5.2). This includes a comparison of the computational efficiency of each model, for both a representative single instance and the complete set of test cases.

### Benchmark solutions

Before introducing the solution benchmarks, it is important to note that, due to hospital practices, each specialty operates within its own designated set of OR blocks (as illustrated in Fig. 1). Patients from one specialty cannot be scheduled into blocks reserved for another, meaning there is no competition for shared blocks across specialties. Consequently, the scheduling problem naturally decomposes by specialty. In our computational approach, we leverage this feature by solving independent subproblems for each specialty separately. Hence, the set $\mathcal {B}$ in our formulation exclusively represents OR blocks associated with a specific specialty for the corresponding week. The proposed two-stage stochastic model and all comparative benchmarks were solved separately for each specialty that is orthopaedics, General, and Urology, and for each day from May to November 2019, resulting in a total of 3×180=540 problem instances.

Each benchmark solution was designed based on how emergency cases and uncertainty were treated, allowing a fair comparison across different decision-making strategies. This setup enables evaluation of the proposed two-stage stochastic model against four benchmark configurations: (i) the current dedicated-policy model, (ii) a deterministic model using expected values, (iii) a deterministic model with perfect information, and (iv) a robust optimisation model. The details of our proposed solution method and all these benchmark solutions, are described in the following subsections.

#### Proposed solution: Flexible ORs with stochastic optimisation (SAA)

The proposed two-stage stochastic model, is solved using the SAA method. SAA replaces the expectation term in the two-stage objective function with an empirical average over a finite set of sampled scenarios, thereby transforming the stochastic problem into a deterministic equivalent while preserving the effects of uncertainty.

For each daily instance, a single mixed-integer program (MIP) is solved that embeds all sampled scenarios within the objective and recourse structure, yielding a sample-average optimal first-stage schedule. This approach captures the expected performance under uncertainty and provides a computationally tractable framework for large-scale hospital scheduling problems.

Formally, the SAA formulation can be expressed as:

20 $$\begin{aligned} \min _{x \in X} \left\{ s^{\top }x + \frac{1}{N} \sum _{n=1}^{N} Q_n(x, \phi _n) \right\} , \end{aligned}$$

Here, x represents the first-stage scheduling decisions, and X denotes the feasible region defined by Constraints Eqs. 1–7. The vector $s^{\top }$ contains the unit costs associated with the first-stage decision variables; hence, $s^{\top }x$ corresponds to the total cost of the first-stage scheduling decisions. $Q_n(x, \phi _n)$ denotes the recourse cost for scenario n given the realization of uncertainty $\phi _n$.

Each of the 540 daily instances was solved using N=500 Monte Carlo–sampled scenarios that jointly reflect the stochastic nature of emergency arrivals and surgery durations. Scenario data were generated from fitted Poisson and lognormal distributions derived from real-world hospital records. The choice of 500 scenarios was based on a trade-off between computational efficiency and solution accuracy—beyond this point, additional scenarios yielded negligible improvement in solution quality while significantly increasing runtime (see Appendix B).

#### Benchmark solution 1: Dedicated ORs (Current Practice)

As the first benchmark, the scheduling outputs were compared against real-world–inspired data from the same period to validate the proposed model using key performance indicators. In the current hospital practice, operating rooms are *dedicated*, meaning that elective and emergency surgeries are scheduled independently in separate rooms, and demand from emergency arrivals is not considered during elective planning. This benchmark therefore represents the hospital’s existing baseline policy, which ignores flexibility in scheduling decisions. The dedicated ORs setup is *observed* rather than solved.

#### Benchmark solution 2: Flexible ORs with deterministic (Expected) values

This benchmark assumes flexible use of operating rooms across both elective and emergency surgeries but fixes the number of daily emergency arrivals and surgery durations at their expected values. It represents an average-case deterministic benchmark that does not explicitly account for uncertainty. Values that are fractional due to averaging have been rounded to their nearest integer to ensure consistency in comparison.

#### Benchmark solution 3: Flexible ORs with deterministic (Perfect Information) values

This benchmark also assumes flexible use of operating rooms across both elective and emergency surgeries but fixes the number of daily emergency arrivals and surgery durations at their perfect information values. In this setting, all random parameters are assumed to be known in advance, representing an idealised scenario in which uncertainty is perfectly predicted. This configuration serves as a deterministic perfect-information benchmark and, like the previous one, does not explicitly account for uncertainty.

#### Benchmark solution 4: Flexible ORs with robust optimisation (CCG)

To evaluate the benefits of robustness, we used a two-stage robust optimisation benchmark inspired by Neyshabouri and Berg [4], solved using the column-and-constraint generation (CCG) framework. The model minimises the worst-case total cost over a budgeted uncertainty set $\mathcal {U}$ constructed from fitted Poisson and lognormal parameters. The master problem determines the first-stage scheduling decisions, while the subproblem identifies the worst-case realisation of uncertainty within $\mathcal {U}$. This approach aims to provide protection against extreme scenarios.

The CCG algorithm iteratively alternates between two optimisation problems:

(i) a master problem, which proposes candidate first-stage decisions under a limited subset of worst-case scenarios, and

(ii) a subproblem, which searches for new extreme realisations of the uncertain parameters that violate the current solution.

New columns and constraints corresponding to these critical scenarios are then added to the master problem until convergence. This decomposition allows efficient solution of large-scale min–max problems without enumerating all uncertainty realisations explicitly.

Formally, the robust optimisation problem can be expressed as:

21 $$\begin{aligned} \min _{x \in X} \left\{ s^{\top } x + \max _{\xi \in \mathcal {U}} Q(x,\xi ) \right\} , \end{aligned}$$

where x represents first-stage scheduling decisions and Q(x,ξ) denotes the recourse cost for uncertainty realisation $\xi \in \mathcal {U}$. Note that the random vector ξ represents the same source of uncertainty as in the SAA formulation, where $\{\phi _n\}_{n=1}^N$ are independent and identically distributed (i.i.d.) realisations of ξ.

**Table 3:** Computational statistics for representative and total problem instances

Policy	Rows	Columns	Average Runtime (s)	Total (540 Days)
Deterministic (Expected Values)	24,870	17,590	6.8	≈ 1.0 h
Deterministic (Perfect Information)	24,870	17,590	6.8	≈ 1.0 h
Stochastic (SAA, *N* = 500)	46,505	34,147	11.9	≈ 2.8 h
Min–Max (CCG)	47,210	34,560	16.3	≈ 4.0 h

*Note: Average runtime refers to a representative daily instance (orthopaedics–1 May 2019)*

Both the SAA-based stochastic and CCG-based robust models share the same two-stage structure: first-stage scheduling decisions are made before uncertainty is realised, and second-stage recourse decisions adapt afterward. In the stochastic formulation (solved via SAA), the second-stage objective is expressed as an expected value over a set of scenarios. In contrast, the robust formulation (solved via CCG) replaces this expectation with a worst-case evaluation, where the model minimises the maximum possible cost over the uncertainty set $\mathcal {U}$, thereby ensuring feasibility and performance guarantees for all $\xi \in \mathcal {U}$.

### Computational performance

This subsection summarises the computational setup and performance of the proposed and benchmark models, including the software environment and the runtime characteristics across problem instances.

#### Computational environment

The proposed stochastic solution, along with all benchmark models—including the robust and deterministic configurations—was implemented and solved using the Gurobi optimiser (version 10.0.3) through its Python interface.

The two-stage stochastic model was solved using the SAA method, in which each sampled realisation of uncertainty yields a deterministic MIP. The optimal first-stage schedule was then selected based on the aggregated performance across all sampled scenarios. Gurobi’s MIP solver efficiently handles large-scale combinatorial optimisation problems through Branch-and-Bound, Cutting Plane, and heuristic strategies.

The robust benchmark was solved using the Column-and-Constraint Generation (CCG) framework [4], which iteratively alternates between optimising first-stage scheduling decisions and identifying worst-case realisations of uncertainty within the uncertainty set $\mathcal {U}$. This procedure was also implemented in Gurobi and does not require explicit enumeration of all scenarios.

#### Runtime and scalability

All optimisation models, including the proposed stochastic formulation, the robust benchmark, and both deterministic configurations, were computationally tractable for the considered hospital-scale instances.

For the two-stage stochastic model solved via the SAA method, a representative daily instance (orthopaedics – 1 May 2019) comprised approximately 46,505 constraints (rows), 34,147 decision variables (columns), and 180,563 nonzero elements in the constraint matrix, requiring about 11.9 seconds to solve. Similar runtimes were observed across other instances, with minor variations due to elective volumes, emergency arrivals, and duration distributions. Solving all 540 daily instances with 500 random scenarios over six months required approximately 2.8 hours in total, confirming the model’s scalability across a realistic hospital planning horizon.

**Table 4:** Adjusted constant parameters by specialty

Parameter	General	Urology	orthopaedics
Regular Block Duration (*R*)	8 hr	8 hr	8 hr
Overtime Limit (*O*)	5 hr	5 hr	5 hr
Cost per Elective Surgery ($$C^a_{ib}$$)	1,050 $	2,150 $	3,250 $
Postponement Cost ($$C^a_{ib'}$$)	2,675 $	3,625 $	4,825 $
Cancellation Cost for Elective Surgery ($$C^c_i$$)	3,580 $	4,670 $	5,520 $
Cost per Emergency Surgery ($$C^a_j$$)	1,580 $	2,680 $	3,780 $
Cancellation Cost for Emergency Surgery ($$C^c_j$$)	8,120 $	9,280 $	10,450 $
Overtime Cost ($$C^o_b$$)	520 $/hr	735 $/hr	915 $/hr

Source: Adapted from Australian Institute of Health and Welfare reports (2019) [36]. Values represent adjusted averages for model application

The robust min–max benchmark, implemented via the Column-and-Constraint Generation (CCG) framework [4], produced slightly larger formulations (≈47,210 rows and 34,560 columns). Each iteration alternated between a master problem—determining first-stage scheduling decisions—and a subproblem—identifying the worst-case realisation within the uncertainty set $\mathcal {U}$. The per-iteration master MIPs retained the same structure as the deterministic model and were solved efficiently by Gurobi. Overall, runtime was moderately higher than that of the SAA model (typically up to 1.5× longer, ≈16.3 seconds per instance).

The deterministic benchmarks were considerably smaller and faster to solve. The expected-value deterministic model contained approximately 24,870 rows and 17,590 columns, with an average runtime of 6.8 seconds per instance. The perfect-information deterministic model had a comparable structure and runtime. However, these models neglect uncertainty and therefore exhibit inferior schedule quality and higher expected costs compared to the stochastic formulation.

Although the proposed two-stage stochastic model includes a large number of variables and constraints, it remains computationally efficient and produces higher-quality, uncertainty-aware schedules. Its average runtime is roughly twice that of the deterministic models, but the improvement in expected scheduling performance makes this increase reasonable. The stochastic formulation also runs faster and is less conservative than the worst-case (CCG-based) optimisation, offering a practical balance between solution quality and computation time. These results (see Table 3) confirm that the proposed model scales well across hundreds of daily instances and remains suitable for real-world hospital applications.

The current-practice configuration (Dedicated ORs) relies on observed hospital data rather than an optimised model and is therefore excluded from the computational comparison. All other benchmark configurations assume flexible allocation of operating rooms between elective and emergency cases.

**Fig. 5:**
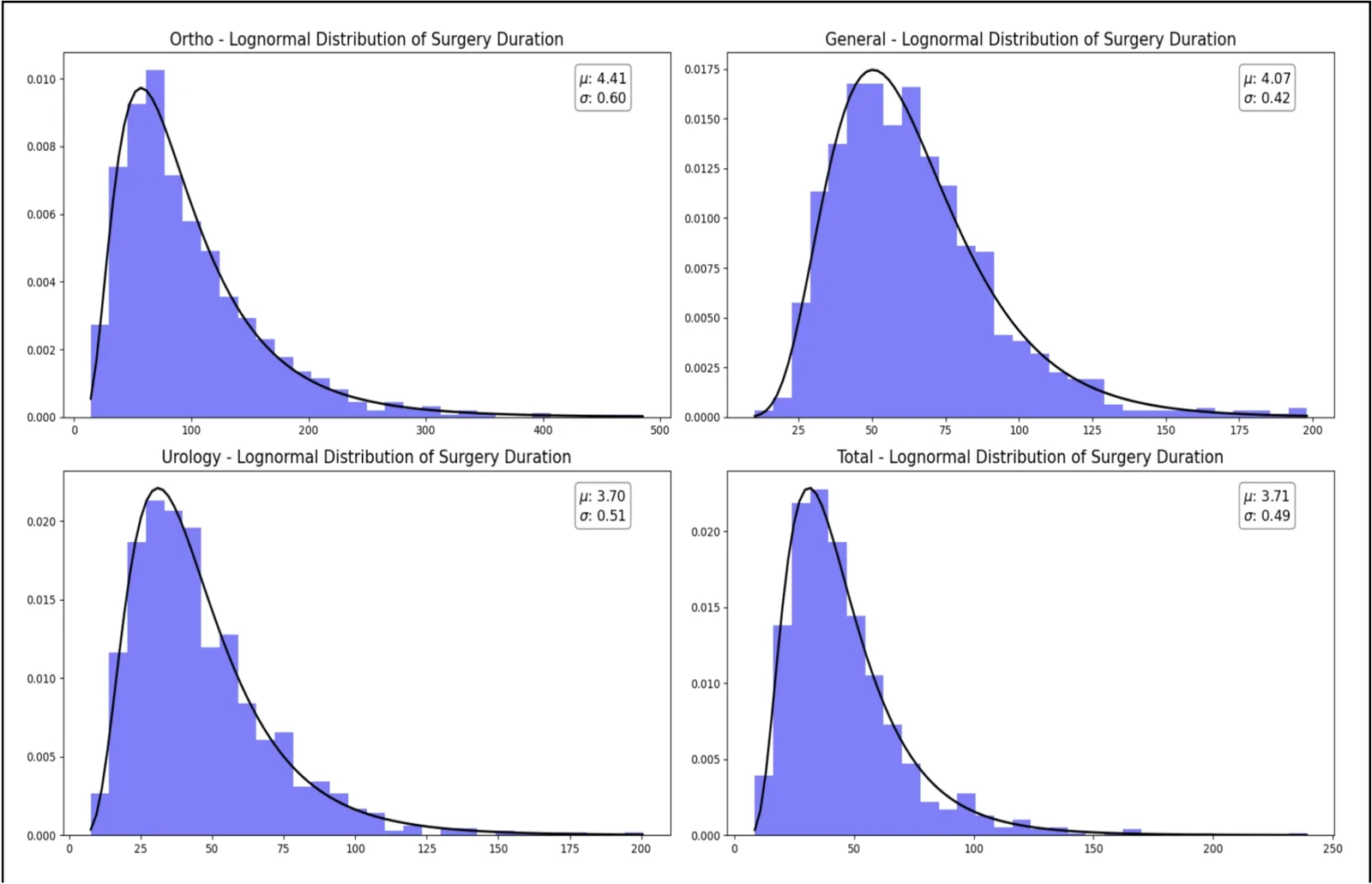
Fitted lognormal distributions for surgery durations by specialty

This comparison highlights that incorporating uncertainty through the proposed two-stage stochastic model enhances scheduling quality, as further demonstrated in the subsequent analysis, while maintaining computational tractability. Overall, these results confirm the model’s scalability and suitability for real-world hospital applications.

#### Model validation

To validate the models, scheduling outputs were compared against real-world-inspired data from the same period, analysing key performance indicators such as the number of elective and emergency surgeries performed, cancellations, OR utilisation rates, and total costs. This comparison confirms that the proposed formulations generate realistic schedules consistent with observed hospital performance patterns.

## Numerical results

This section presents the numerical results obtained from implementing the proposed two-stage stochastic model, solved via the SAA method, along with the benchmark solutions introduced in Section 5. The analysis focuses on key performance metrics, including surgery cancellations, total costs, OR utilisation rates, and related indicators.

All models were evaluated using hospital-inspired data from May to November 2019, with the current-practice configuration (Dedicated ORs) serving as the baseline for comparison. The experiments were conducted for three surgical specialties—General Surgery, orthopaedics, and Urology—across weekdays (Monday to Friday). The process used to construct the synthetic dataset is detailed in the Appendix A.

Finally, to assess model robustness, a comprehensive sensitivity analysis was performed at the end of the numerical evaluation. The analysis investigates how total costs, cancellations, and operating-room utilisation respond to variations in key parameters, including the emergency arrival rate λ, the mean and standard deviation of surgery durations, and selected cost coefficients. These results provide valuable insights into the stability of the proposed scheduling model and the influence of uncertainty and cost structures on overall system performance.

### Model settings

This subsection outlines the configuration of the experimental setup, including model parameters, random scenario generation, and the uncertainty set employed in the robust benchmark. These elements establish the foundation for evaluating how the proposed model captures variability and optimises surgical schedules under uncertainty.

#### Model parameters

The model parameters are categorised into two groups: **constant** and **uncertain** components. Constant parameters were either obtained directly from hospital data or calibrated using institutional reports, whereas uncertain parameters were modelled differently under the stochastic and robust frameworks, based on synthetic distributions inspired by real-world data.

The dataset used in this study corresponds to an Australian hospital-inspired dataset covering the period from May to November 2019. This time window aligns with the tactical scheduling plan defined by the master surgery schedule shown in Fig. 1, ensuring that the higher-level allocation of operating rooms remains consistent across all models and experiments.

##### Constant parameters

Table 4 summarizes the main fixed parameters, including regular block durations, overtime limits, and cost coefficients. These values remained constant across all scenarios and benchmark solutions and were defined separately for each specialty.

**Fig. 6:**
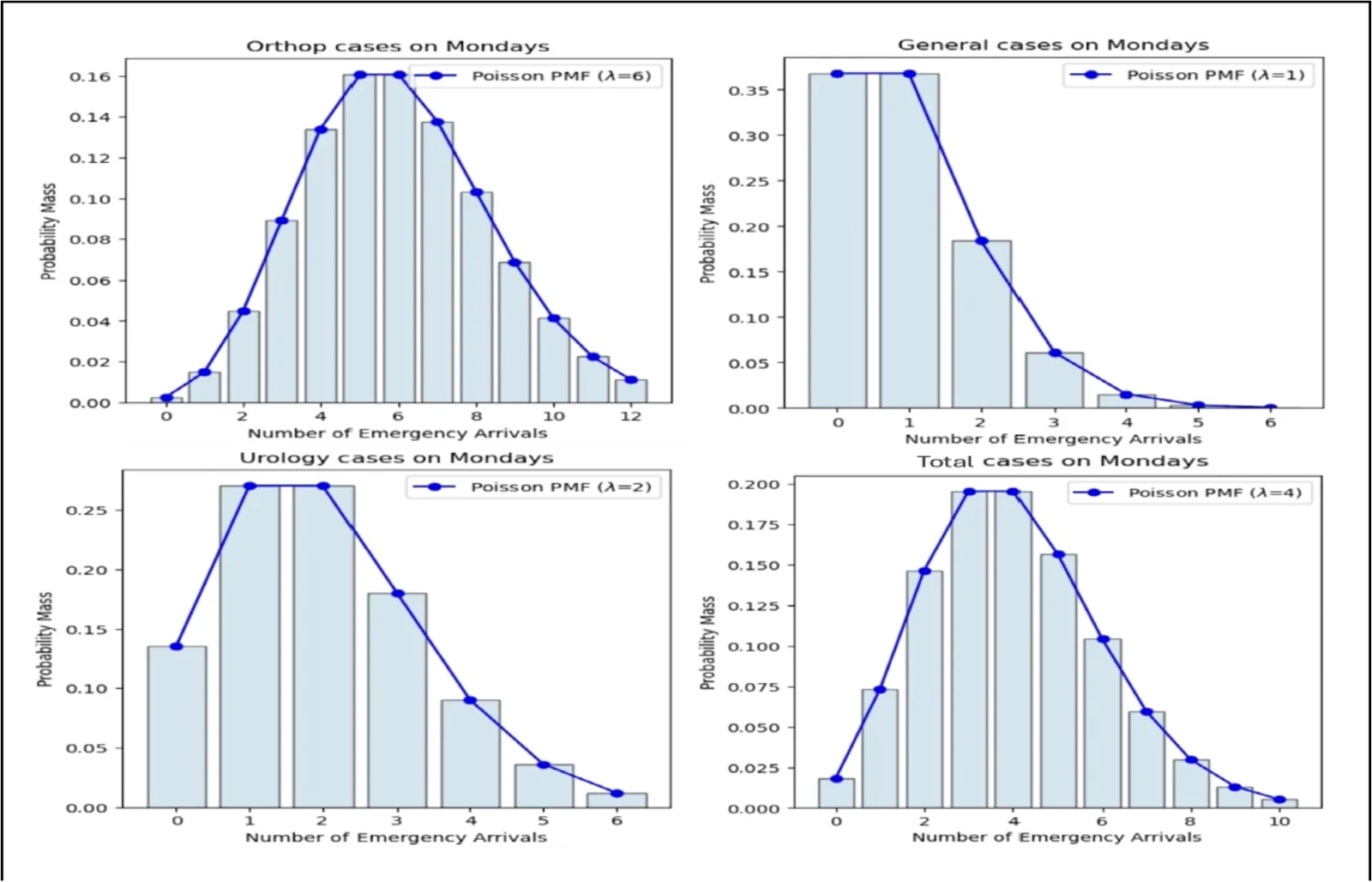
Poisson distributions of emergency case arrivals by specialty

##### Uncertain parameters

The primary sources of uncertainty in the model are surgery durations and emergency arrivals. These parameters were characterised differently under the stochastic and robust frameworks to ensure a consistent yet comparable treatment of uncertainty.

**(a) Stochastic characterisation.**

In the proposed two-stage stochastic model, uncertainty is represented by probability distributions derived from hospital-inspired data. Surgery durations were best fitted with a *Lognormal distribution*, which is commonly used for right-skewed healthcare data [11, 12, 14]. In this distribution, the parameters μ and σ denote the *location* and *shape* parameters, corresponding to the mean and standard deviation of the log-transformed surgery durations, respectively. According to Fig. 5, Orthopaedic surgeries exhibited the highest fitted parameters (μ=4.41, σ=0.60), followed by General (μ=4.07, σ=0.42) and Urology (μ=3.70, σ=0.51). For instance, in the Orthopaedic specialty, these fitted parameters yield an expected surgery duration of $E[X] = e^{4.41 + 0.5(0.6)^2} \approx 99$ minutes, where *X* denotes the *surgery duration* (in minutes). This demonstrates how the lognormal model effectively captures the positively skewed nature of surgical time distributions.

Emergency arrivals were modelled as a Poisson process, consistent with common assumptions in healthcare operations research [12, 17–19]. Because emergency arrival patterns vary by day of the week, weekday arrivals were modelled separately. Figure 6 illustrates the distribution of emergency arrivals for each specialty on Mondays, based on the synthetic dataset. As shown, the specialty-specific mean arrival rates were λ=1 (General), λ=6 (orthopaedics), and λ=2 (Urology).

**(b) Robust characterisation.**

For the robust benchmark, the same uncertain parameters—surgery durations and emergency arrivals—were represented within a bounded uncertainty set instead of probability distributions. Following Neyshabouri and Berg [4], a budgeted uncertainty set was used to limit how many parameters can deviate from their nominal values at the same time:

22 $$\begin{aligned} \mathcal {U} = \Big \{ \xi = \bar{\xi } + \eta \circ \delta \;\Big |\; -1 \le \eta _k \le 1,\; \sum _k |\eta _k| \le \Gamma \Big \}. \end{aligned}$$

Here, $\bar{\xi }$ denotes the nominal (mean) values, and δ defines the maximum allowed deviations, set to 25% of each nominal value. The vector $\eta = (\eta _1, \eta _2, \ldots , \eta _{n_\xi })$ contains deviation multipliers, where each $\eta _k \in [-1,1]$ adjusts the *k*th uncertain parameter. The index *k* simply refers to each uncertain parameter, and $n_\xi$ is the total number of them (for example, different surgery durations and emergency arrival rates). The symbol ∘ denotes the elementwise (Hadamard) product, meaning that each parameter varies independently as $\xi _k = \bar{\xi }_k + \eta _k \delta _k$. The uncertainty budget Γ controls how many parameters can deviate at once: Γ=0 gives the deterministic case, while larger Γ values lead to more conservative solutions. This structure allows a fair comparison between the two-stage stochastic model and its robust counterpart.

**Fig. 7:**
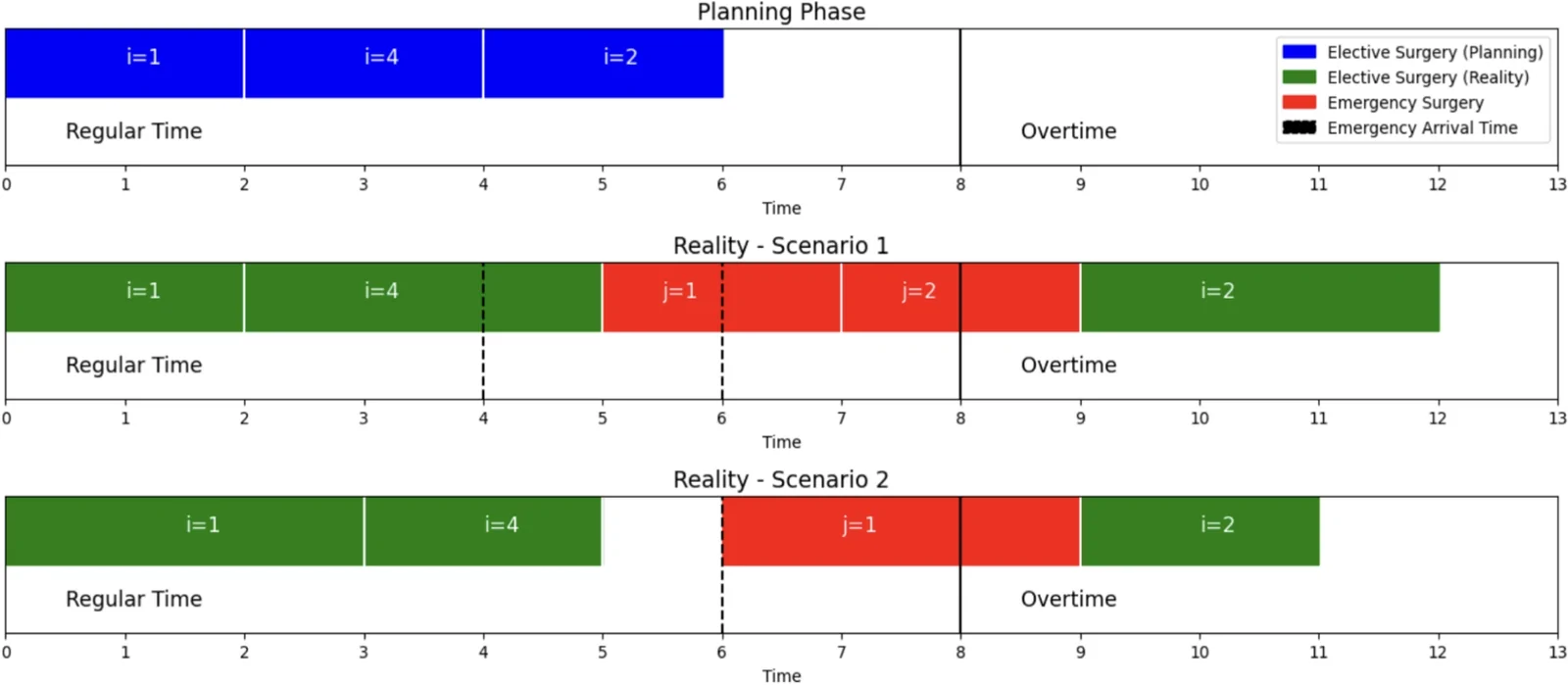
Scheduling for Urology–Wednesday–OR 02

#### Performance metrics

To evaluate the model’s effectiveness, several key performance metrics were defined to capture both surgical outcomes and OR resource utilisation:

- **Performed elective surgeries:** The number of performed elective surgeries measures how successfully scheduled elective procedures are completed within available OR blocks, reflecting the system’s ability to utilise elective capacity efficiently while reducing the elective waiting list.
- **Performed emergency surgeries:** The number of performed emergency surgeries indicates how effectively the model accommodates urgent cases while minimising disruptions to planned elective operations.
- **Postponed elective surgeries:** The number of postponed elective surgeries represents cases that are deferred at the beginning of the week due to resource constraints.
- **Cancelled elective surgeries:** Cancelled elective surgeries occur on the day of surgery (the second stage of the model) when elective procedures are cancelled because of unexpected emergency arrivals or limited OR availability.
- **Cancelled emergency surgeries:** Cancelled emergency surgeries occur when urgent cases cannot be accommodated in time, which can compromise patient safety and reflect system-level inefficiencies in hospital performance.
- **Total cost ($):** The total cost captures all expenses associated with performed surgeries, overtime, postponements and cancellations, providing an overall measure of the system’s financial efficiency.
- **Average OR utilisation (%):** The average OR utilisation represents the proportion of total operating room time actively used for surgeries. Higher utilisation reflects more efficient use of resources, less idle time, and better coordination of surgical activities.

**Fig. 8:**
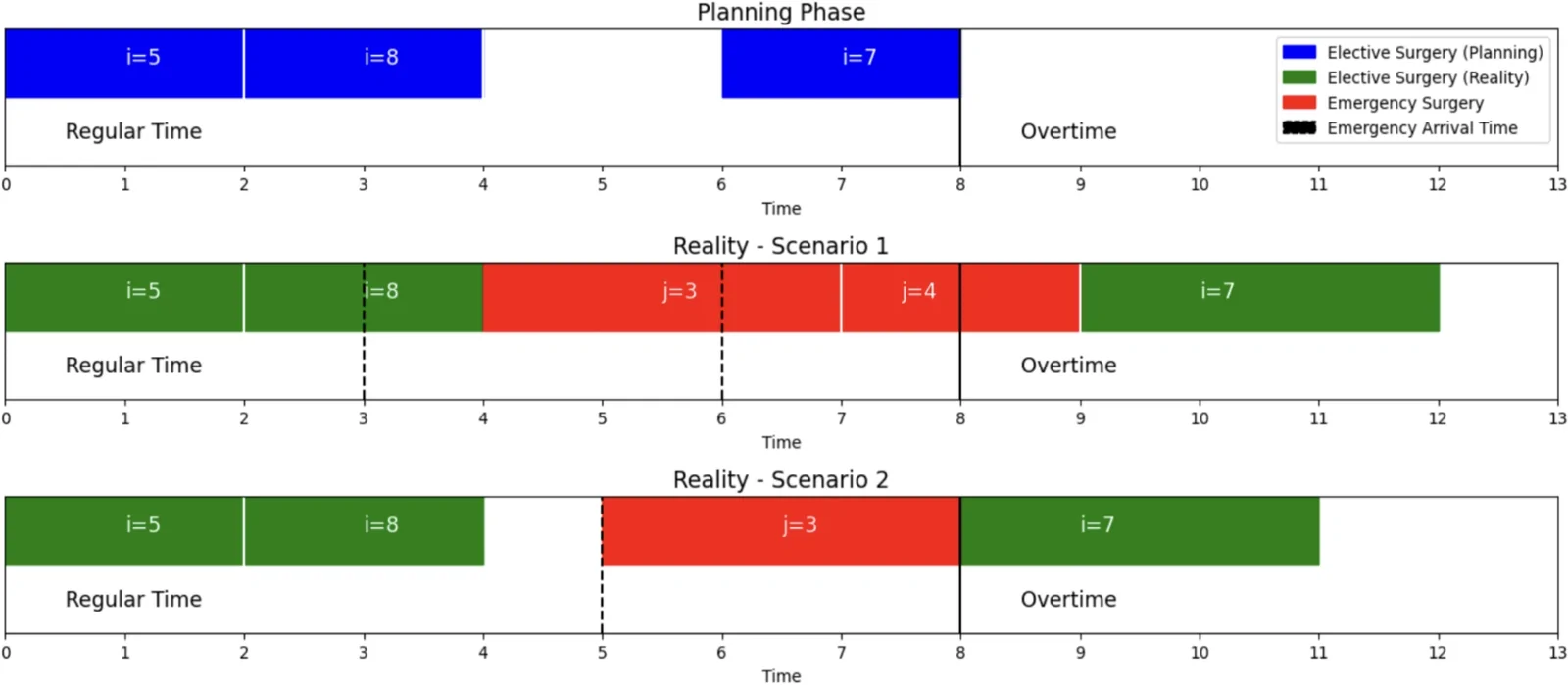
Scheduling for Urology–Wednesday–OR 12

**Fig. 9:**
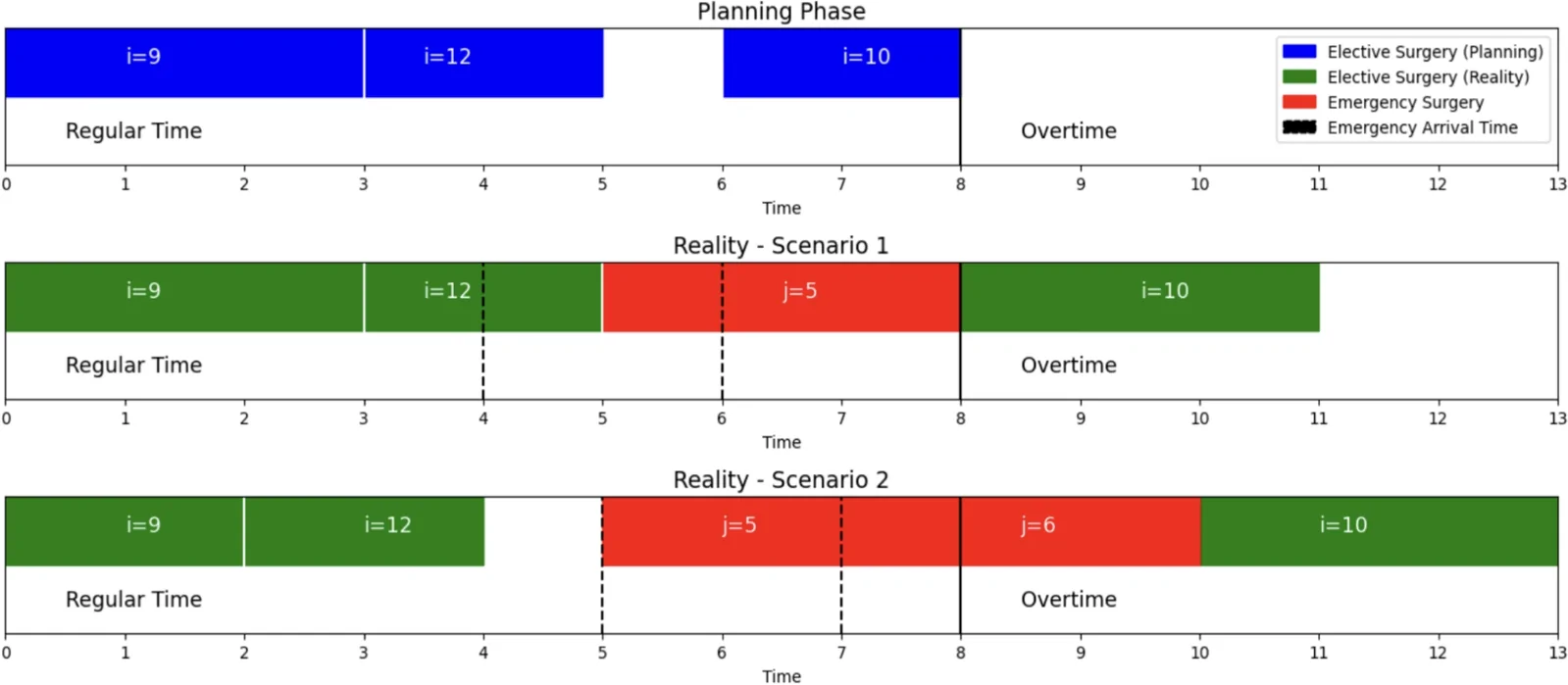
Scheduling for Urology–Thursday–OR 02

#### Scenarios

To capture the uncertainty inherent in daily OR operations, a set of 500 random scenarios was generated to reflect variability in both surgery durations and emergency arrivals. These scenarios were constructed using hospital synthetic data from May to November 2019, ensuring a realistic representation of operational conditions.

In the two-stage stochastic model, each scenario corresponds to a distinct realisation of the uncertain parameters and forms the basis of the Sample Average Approximation framework. The SAA method evaluates each candidate scheduling decision across all generated scenarios, allowing the model to identify a solution that performs well on average under uncertainty.

Scenario generation integrates both sources of randomness: surgery durations are modelled using a lognormal distribution, while emergency arrivals follow a Poisson process. This combination reproduces the stochastic behaviour typically observed in real surgical environments, enabling robust and data-driven performance evaluation.

### A case study

This section presents a case study illustrating the application of the proposed two-stage stochastic scheduling model for allocating surgeries to OR blocks over a specific week. The example focuses on the Urology department, which had 16 elective surgery cases scheduled for the week beginning Sunday, June 9, 2019.

During this week, the department was assigned four surgical blocks for Urology procedures. Since Monday was a public holiday, surgeries were scheduled from Tuesday to Friday, with two ORs operating on both Wednesday and Thursday (see Fig. 1). The scheduling process involved assigning 16 elective surgeries across these blocks while maintaining flexibility for potential emergency arrivals. Regular work hours were set at 8 hours per day, with up to 5 additional hours permitted as overtime. Emergency cases had a maximum allowable waiting time of 2 hours; if not accommodated within this limit, they were cancelled and potentially transferred to another hospital. Urology surgery durations followed a lognormal distribution with parameters μ=3.70 and σ=0.51 (in hours).

To analyse the model’s response to uncertainty, two representative scenarios were considered. The first represents a high emergency-load situation with frequent unplanned arrivals, while the second depicts a more balanced scenario with moderate emergency demand. These two cases demonstrate the model’s adaptability in managing unpredictable emergency arrivals while maintaining elective scheduling stability.

**Fig. 10:**
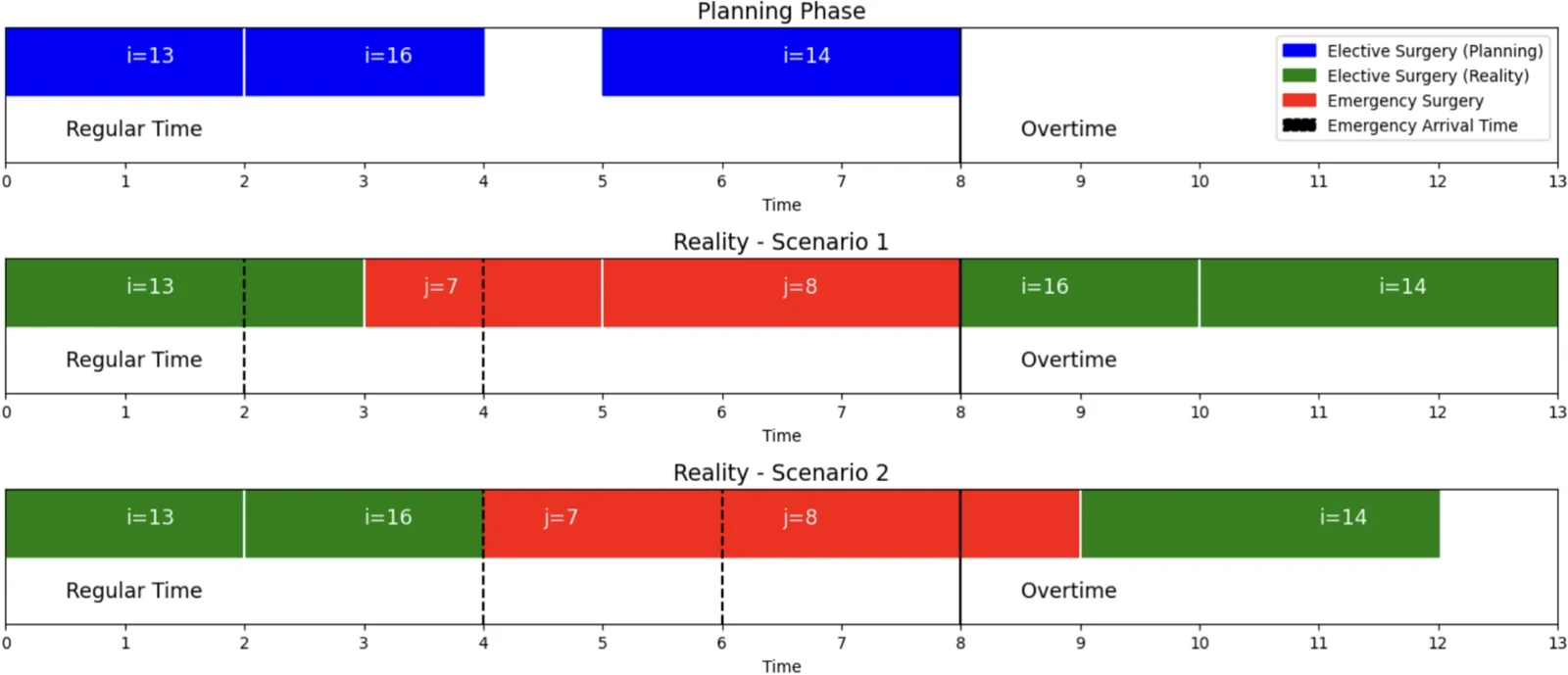
Scheduling for Urology–Thursday–OR 12

Figures 7, 8, 9 and 10 illustrate how surgeries were allocated across the four available blocks, comparing the planned (first-stage) schedule with realised (second-stage) schedules under both scenarios.

During the planning phase (first stage), buffer times were intentionally left within each block to accommodate potential emergency arrivals, preventing elective surgeries from occupying high-risk time slots. For example, in Fig. 7, surgery i=2 could not start at t=5 because the interval from t=6 to t=8 was reserved for emergencies. Allowing an elective surgery in this period would increase the likelihood of overlap in the event of an emergency.

At the beginning of the week, elective surgeries i=3, i=6, i=11, and i=15 were postponed to the following week’s schedule based on the predicted emergency arrivals (expected value of second-stage costs). In the realised scenarios, we also observe that some elective cases are deferred within the same day to allow sufficient time for emergency surgeries that must be performed within their limited waiting periods. This demonstrates how the model dynamically adjusts elective scheduling decisions to accommodate unforeseen emergency arrivals.

Overall, this case study illustrates the model’s ability to balance elective and emergency surgeries by maintaining planned buffer times and adapting schedules as uncertainty unfolds. These adaptive adjustments help reduce cancellations and emergency delays, improving both operating room utilisation and overall resource efficiency. However, it is good to mention that relying solely on the BIM policy (see the top panel of Fig. 4) yields a suboptimal strategy when urgent-case demand is highly uncertain. Its fixed insertion opportunities cannot fully capture stochastic variability, often leading to emergency delays or cancellations—issues that the proposed buffer-based optimisation model addresses more effectively.

### Performance analysis

Table 5 reports the comparative performance of the five scheduling models introduced in Section 5, evaluated across three surgical specialties—General Surgery, orthopaedics, and Urology—over the period from May to November 2019. These include the current practice with dedicated ORs, the deterministic flexible setup using expected parameter values, deterministic flexible setup using perfect information values, and two flexible policies that explicitly handle uncertainty—the proposed two-stage stochastic model solved via SAA and the robust benchmark based on a budgeted uncertainty set (Neyshabouri and Berg [4]).

All models were evaluated using performance metrics averaged over (N=50) Monte Carlo scenarios per week, where each scenario corresponds to an independent realisation of surgery durations and emergency arrivals.. The reported figures represent weekly averages computed from approximately 540 daily instances, providing a statistically consistent basis for comparison (from May to November 2019).

The analysis focuses on the key performance metrics introduced in Section 6.1.2.

Here, the final column reports the relative percentage change of the proposed stochastic policy compared with the current practice (Dedicated ORs), computed as

23 $$\begin{aligned} \%\Delta = \frac{\text {Stochastic} - \text {Dedicated}}{\text {Dedicated}} \times 100\%. \end{aligned}$$

Positive values indicate improvement (e.g., higher utilisation or more surgeries performed), while negative values denote reductions in undesirable outcomes such as costs or cancellations. This column therefore quantifies the performance gain of the proposed two-stage stochastic model relative to current hospital scheduling practice.

Overall, the results in Table 5 show that the proposed two-stage stochastic model consistently outperforms the current practice with dedicated ORs across nearly all key performance indicators. While the number of performed elective surgeries decreases slightly (–3.1%) due to the prioritisation of emergencies, the number of performed emergency surgeries increases markedly (+12.1%), indicating improved responsiveness to urgent demand. Cancellations show a clear downward trend—elective cancellations decrease by 4.7% and emergency cancellations by 26.2%—highlighting enhanced scheduling stability under uncertainty. Total operating costs drop by approximately 18.3%, and average OR utilisation improves by 7.7%, from 78.2% to 84.2%.

The two-stage stochastic model performs almost as well as the deterministic perfect-information case, which represents the ideal scenario where all emergency arrivals and surgery durations are known in advance. Across all specialties, the total cost difference between the two is less than 1% ($2.48M vs. $2.54M), showing that the proposed model achieves near-optimal performance without needing complete knowledge of future events.

**Table 5:** Dedicated vs Flexible ORs with certain and uncertain emergency modelling (weekly averages; May–Nov 2019)

		Dedicated ORs	Flexible ORs				%Δ Stoch vs Ded
Specialty	Metric	Current Practice	Det. (Expected)	Det. (Perfect Info.)	Stochastic (Proposed)	Robust	
General	Performed Elective	46.2±4.0	45.0±3.8	44.8±3.2	$$\mathbf {44.3\pm 3.0}$$	43.0±3.5	-4.11%
	Performed Emergency	37.5±4.0	37.8±4.2	39.5±4.0	$$\mathbf {39.2\pm 4.0}$$	38.5±4.1	+4.53%
	Postponed Elective	36.1±5.0	37.2±4.8	37.5±4.6	$$\mathbf {38.1\pm 5.0}$$	39.5±4.9	+5.54%
	Cancelled Elective	18.2±4.0	17.6±3.7	16.8±3.2	$$\mathbf {17.2\pm 3.0}$$	18.5±3.3	-5.49%
	Cancelled Emergency	16.2±5.0	16.0±4.6	14.8±2.5	$$\mathbf {15.1\pm 2.0}$$	16.0±2.3	-6.79%
	Total Costs ($)	923,454 ±33,200	860,000 ±30,000	810,000 ±25,000	**825,318** **±26,500**	845,000 ±28,000	-10.62%
	Average Utilisation (%)	81.3±6	84.5±6	87.0±5	$$\mathbf {86.2\pm 5}$$	85.0±5	+6.03%
Orthopaedic	Performed Elective	38.5±2.0	39.0±2.5	40.5±2.7	$$\mathbf {40.1\pm 3.0}$$	38.0±2.6	+4.16%
	Performed Emergency	33.4±3.0	37.0±3.5	41.8±4.5	$$\mathbf {41.2\pm 5.0}$$	39.0±4.2	+23.35%
	Postponed Elective	32.1±4.0	31.0±3.8	29.0±3.4	$$\mathbf {29.2\pm 3.0}$$	31.5±3.6	-9.03%
	Cancelled Elective	16.2±2.0	15.0±1.9	13.9±1.8	$$\mathbf {14.2\pm 2.0}$$	15.8±2.1	-12.35%
	Cancelled Emergency	17.1±3.0	12.0±2.5	8.8±1.8	$$\mathbf {9.1\pm 2.0}$$	10.5±2.2	-46.20%
	Total Costs ($)	1,123,465 ±19,000	980,000 ±22,000	910,000 ±18,000	**932,545** **±15,200**	965,000 ±18,000	-16.98%
	Average Utilisation (%)	74.2±3	80.0±4	85.0±4	$$\mathbf {84.1\pm 4}$$	82.5±4	+13.34%
Urology	Performed Elective	32.5±3.0	30.0±2.3	29.8±2.1	$$\mathbf {29.2\pm 2.0}$$	28.5±2.2	-10.15%
	Performed Emergency	30.3±2.0	31.0±2.4	33.4±2.8	$$\mathbf {33.1\pm 3.0}$$	31.5±2.7	+9.24%
	Postponed Elective	41.3±3.0	43.0±3.0	43.8±3.1	$$\mathbf {44.1\pm 3.0}$$	45.5±3.1	+6.78%
	Cancelled Elective	12.3±1.0	12.8±1.0	12.5±0.9	$$\mathbf {13.1\pm 1.0}$$	13.8±1.1	+6.50%
	Cancelled Emergency	13.3±3.0	11.5±2.2	9.8±1.2	$$\mathbf {10.2\pm 1.0}$$	11.0±1.2	-23.31%
	Total Costs ($)	1,024,534 ±21,200	880,000 ±18,000	820,000 ±17,000	**842,178** **±18,100**	865,000 ±19,000	-17.82%
	Average Utilisation (%)	79.3±3	81.0±2	83.0±1	$$\mathbf {82.4\pm 1}$$	81.5±2	+3.91%
Total	**Total Performed Elective**	117.2±10.0	114.0±10.5	115.1±9.5	$$\mathbf {113.6\pm 9.0}$$	109.5±9.6	$$\mathbf {-3.07\%}$$
	**Total Performed Emergency**	101.2±10.0	105.8±11.0	114.7±11.5	$$\mathbf {113.5\pm 12.0}$$	110.0±11.5	$$\mathbf {+12.12\%}$$
	**Total Postponed Elective**	109.5±13.0	111.2±12.0	110.3±11.0	$$\mathbf {111.4\pm 10.0}$$	116.5±11.0	$$\mathbf {+1.73\%}$$
	**Total Cancelled Elective**	46.7±5.0	45.4±4.8	44.0±4.2	$$\mathbf {44.5\pm 4.0}$$	48.1±4.5	$$\mathbf {-4.71\%}$$
	**Total Cancelled Emergency**	46.6±10.0	39.5±9.5	33.2±6.0	$$\mathbf {34.4\pm 6.0}$$	36.0±6.8	$$\mathbf {-26.18\%}$$
	**Total Costs ($)**	3,095,854 ±82,500	2,720,000 ±68,000	2,480,000 ±62,000	**2,529,463** **±61,200**	2,675,000 ±70,000	$$\mathbf {-18.28\%}$$
	**Total Avg.** **Utilisation (%)**	78.2±12	82.0±11	85.0±10	$$\mathbf {84.2\pm 11}$$	83.0±11	$$\mathbf {+7.67\%}$$

**Notes.** *Dedicated ORs* represent current practice (separate elective/emergency rooms). Under *Flexible ORs*, *Det. (Expected)* fixes emergency arrivals at expected values; *Det. (Perfect Info.)* assumes perfect information about all random parameters; *Stochastic (Proposed)* applies the two-stage SAA model; and *Robust* solves a worst-case (min–max) benchmark. The final column shows the percentage change of the *Stochastic (Proposed)* policy relative to *Dedicated ORs*: $\% \Delta = \frac{\text {Stochastic} - \text {Dedicated}}{\text {Dedicated}} \times 100\%$. Values are weekly means ± standard deviations across weeks (May–Nov 2019)

When compared with the deterministic expected-value model, the improvements are much clearer. Total costs drop by around 7% ($2.72M to $2.53M), emergency cancellations decrease by about 13%, and operating-room utilisation increases from 82% to 84%. These gains demonstrate the benefit of explicitly modelling uncertainty rather than relying on average estimates, which tend to underestimate day-to-day variability.

Compared with the robust benchmark, the stochastic approach is less conservative and more efficient. It reduces total costs by roughly 6% ($2.68M vs. $2.53M) and achieves slightly higher utilisation (84.2% vs. 83%), offering a more balanced trade-off between efficiency and preparedness. Overall, the two-stage stochastic model provides a realistic and well-performing scheduling strategy that balances cost, reliability, and responsiveness under uncertainty.

#### Performance metrics for stochastic and robust scheduling decisions

To evaluate how uncertainty affects scheduling decisions, we report two standard indicators widely used in stochastic optimisation: the Value of the Stochastic Solution (VSS) and the Price of Robustness (PoR).

The VSS measures the benefit of explicitly accounting for uncertainty and is defined as

24 $$\begin{aligned} \text {VSS} = \mathbb {E}_{\xi }\!\left[ C(x_{\text {EV}}, \xi ) \right] - \mathbb {E}_{\xi }\!\left[ C(x_{\text {SAA}}, \xi ) \right] , \end{aligned}$$

where $x_{\text {EV}}$ is the deterministic *expected-value* solution (obtained by fixing random parameters, such as emergency arrivals, at their mean values), and $x_{\text {SAA}}$ denotes the solution of the proposed two-stage stochastic model. In a minimisation context, $\text {VSS} \ge 0$ represents the expected cost savings achieved by explicitly modelling uncertainty rather than relying on deterministic averages.

The PoR quantifies the cost premium of adopting a robust solution that guards against worst-case outcomes:

25 $$\begin{aligned} \text {PoR} = \frac{ \mathbb {E}_{\xi }\!\left[ C(x_{\text {ROB}}, \xi ) \right] - \mathbb {E}_{\xi }\!\left[ C(x_{\text {SAA}}, \xi ) \right] }{ \mathbb {E}_{\xi }\!\left[ C(x_{\text {SAA}}, \xi ) \right] } \times 100\%. \end{aligned}$$

Here, $x_{\text {ROB}}$ represents the robust solution optimised under a budgeted uncertainty set, while $x_{\text {SAA}}$ again corresponds to the stochastic policy. A positive PoR indicates that the robust approach trades a small increase in expected cost for improved protection against extreme scenarios.

To further analyse these trade-offs, both metrics were computed for each specialty. Positive VSS values indicate that the two-stage stochastic model achieves lower expected costs compared with deterministic planning, while PoR measures the additional cost of ensuring robustness. Numerically, VSS remained positive across all specialties (overall: 18.600 $ [95% CI: 15.400 $–21.700 $]), and the robust policy incurred a modest PoR of 3.9 %  [95% CI: 3.2 %–4.6 %], confirming the expected trade-off between average-case efficiency and worst-case reliability.

A higher VSS indicates that explicitly accounting for uncertainty leads to better cost efficiency compared with deterministic scheduling. In contrast, the moderate PoR values show that although the robust model maintains feasibility under worst-case conditions, it does so at a higher cost and with lower efficiency than the stochastic approach. Overall, as shown in Table 6, the proposed two-stage stochastic model achieves a practical balance between cost efficiency and solution reliability under uncertainty.

**Table 6:** Value of the Stochastic Solution (VSS) and Price of Robustness (PoR)

Specialty	VSS ($)	PoR (%)
General	14.800	3.3
orthopaedics	21.500	4.2
Urology	19.500	3.8
Total (avg.)	18.600	3.9

**Fig. 11:**
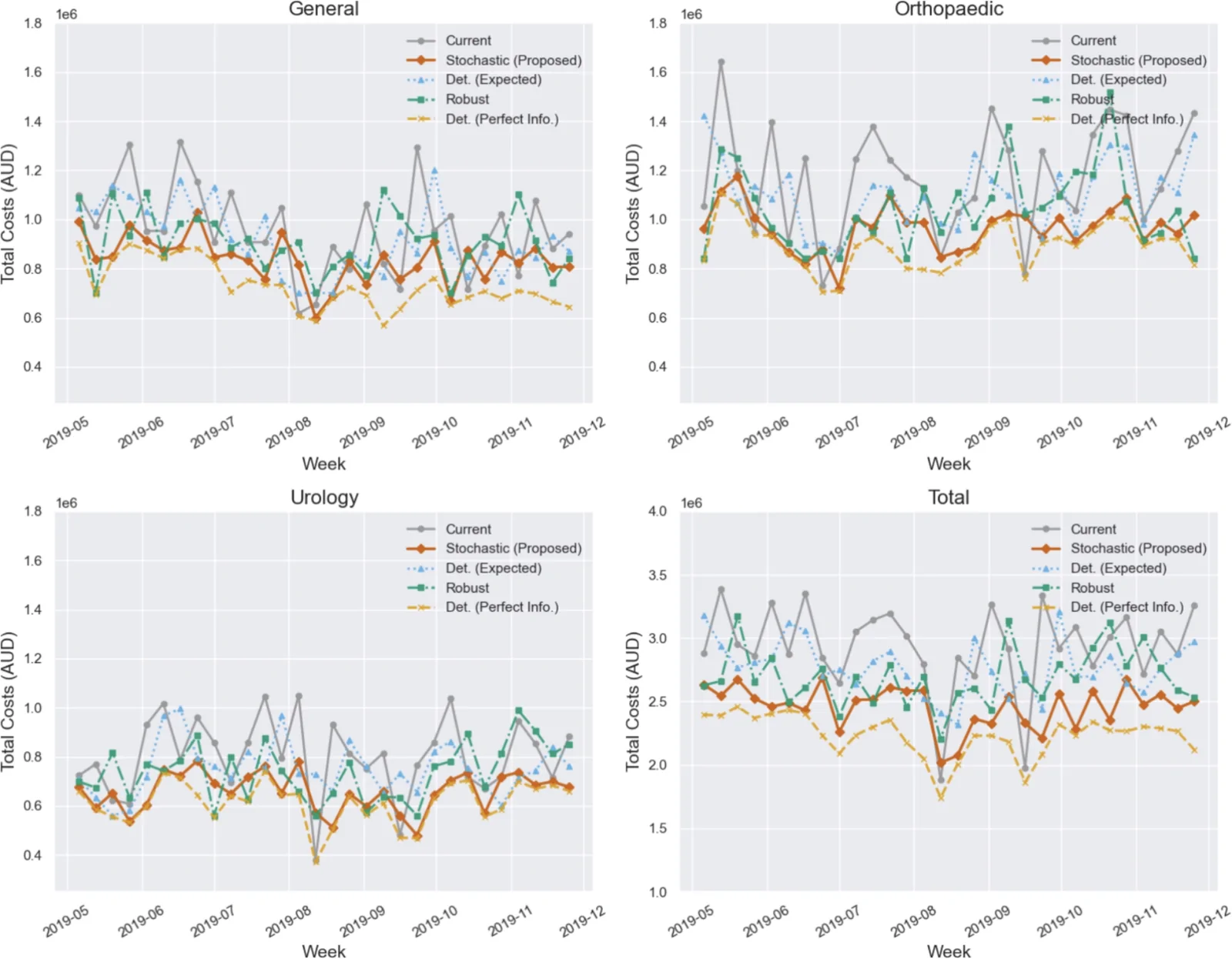
Time series of total costs ($) by week (May to November 2019)

### Trends in performance metrics over time

This section examines temporal trends in key performance metrics from May to November 2019. By analyzing time series data for total costs, emergency cancellations, and average utilisation, we assess the proposed two-stage stochastic model’s adaptability to fluctuations in surgical demand and its impact on OR operations. Comparisons are also made with other scheduling models.

#### Total costs over time

Figure 11 shows the weekly evolution of total costs across specialties under five scheduling models: the current dedicated-room practice, deterministic flexible scheduling using expected values, deterministic flexible scheduling using perfect information, the proposed two-stage stochastic model, and the robust benchmark. The trends highlight clear differences in variability and performance across specialties.

For General Surgery, the current practice shows the largest week-to-week fluctuations, possibly driven by irregular emergency arrivals and overtime use. The deterministic policy smooths some of this variation but remains sensitive to demand changes. The two-stage stochastic model keeps costs consistently lower and more stable, while the robust policy lies slightly above it due to its conservative design. A few weeks (e.g., mid-August) show short-term cost increases in the stochastic results, likely reflecting temporary variability in emergency arrivals or case durations. Overall, the results obtained by the two-stage stochastic model closely match the perfect-information benchmark, indicating that it captures the main effects of uncertainty effectively.

For orthopaedics, the two-stage stochastic model performs best overall, delivering substantial cost reductions with minimal variation. Occasional peaks—such as in mid-June and late September—show stochastic costs briefly higher than current practice, possibly from overestimated emergency arrivals or unexpectedly long surgeries. The deterministic policy reduces costs on average but still exhibits strong week-to-week swings. The robust policy closely follows the stochastic results but remains slightly higher due to its built-in caution against worst-case events. Likewise, the stochastic results remain near the perfect-information benchmark, performing almost as well as if future conditions were known.

For Urology, both the stochastic and robust models yield smoother and lower cost patterns than the current or deterministic policies. Minor short-term increases in the stochastic series—such as in July and September—occur when emergency demand exceeded expectations, leading to brief overtime use. Despite these deviations, the two-stage stochastic model remains the most stable and cost-efficient overall. Its path lies just above the perfect-information case, capturing nearly all attainable efficiency gains under uncertainty.

Across all specialties, the two-stage stochastic model achieves low and stable costs, nearly matching the perfect-information outcome. The robust policy offers a cautious but steady alternative, while the deterministic model improves on current practice yet stays sensitive to variation. Overall, modeling uncertainty improves stability, predictability, and cost efficiency.

**Fig. 12:**
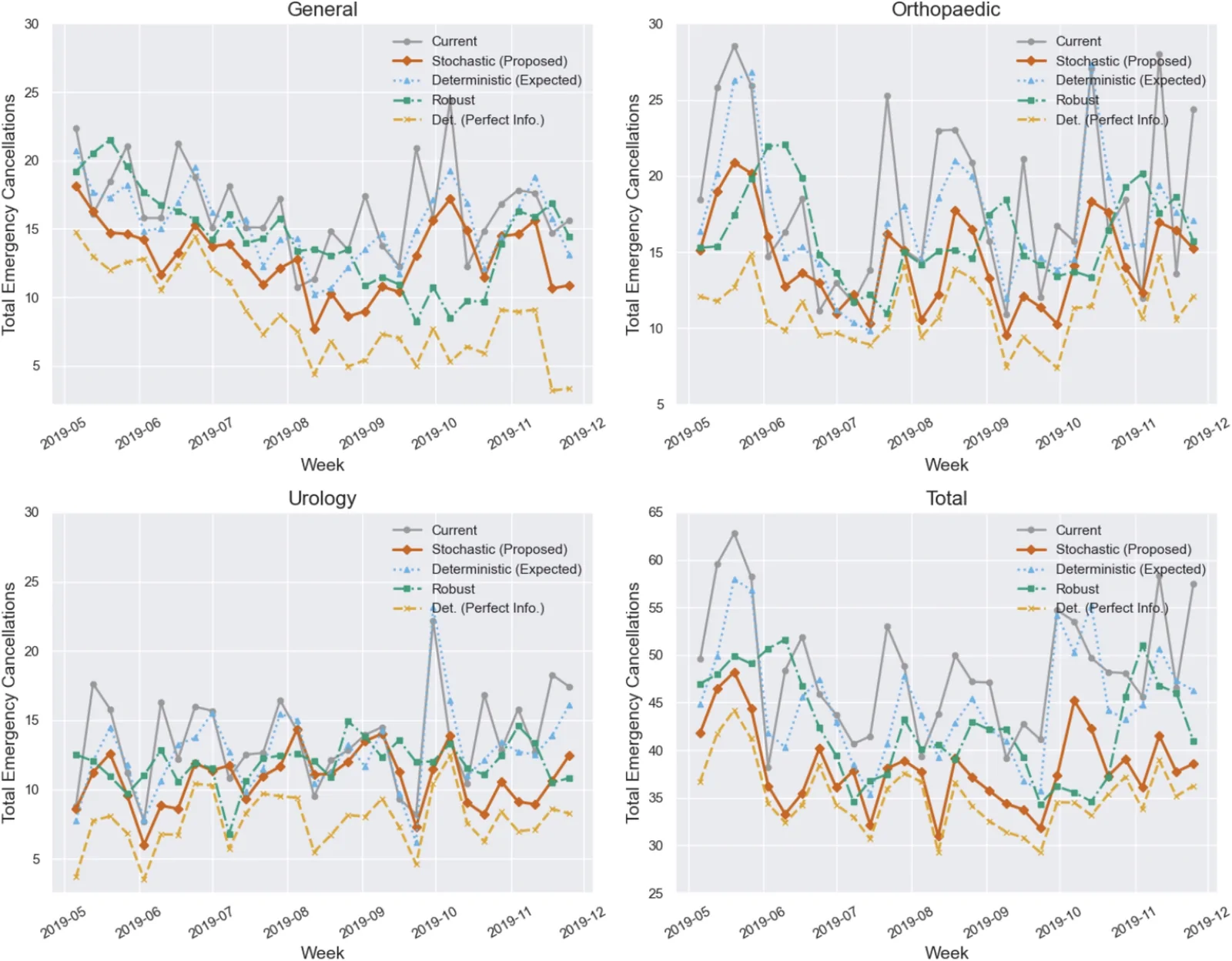
Time series of emergency cancellations by week (May to November 2019)

#### Emergency cancellations over time

Figure 12 compares weekly emergency surgery cancellations across specialties under five scheduling models. The patterns demonstrate how accounting for uncertainty influences performance and stability in handling last-minute disruptions.

For General Surgery, the current system exhibits marked week-to-week variation, with peaks around June and October. Both deterministic (expected) and robust policies reduce these surges, while the two-stage stochastic model achieves consistently lower and smoother cancellation rates throughout the period. The perfect-information benchmark lies slightly below all others, serving as a theoretical lower bound that the stochastic line closely approaches—indicating that uncertainty has been effectively captured. Small temporary rises in the stochastic curve, such as mid-July, reflect transient mismatches between predicted and actual emergency arrivals.

For orthopaedics, the current practice remains highly erratic, with sharp spikes in May, July, and October. Deterministic (expected) scheduling smooths these fluctuations but still tracks demand swings. The robust model further stabilises performance yet occasionally rises during high-pressure weeks. The two-stage stochastic model again achieves the lowest and most stable cancellation levels, nearly aligning with the perfect-information trajectory—confirming its strong adaptability under uncertainty.

For Urology, scattered peaks in July and October may stem from complex cases or temporary operating-room bottlenecks. All model-based strategies outperform the current practice, with the stochastic policy maintaining the lowest and most consistent cancellation pattern. The perfect-information line remains marginally better, as expected, highlighting the closeness of the stochastic results to the ideal optimum.

Across all specialties, the two-stage stochastic model produces the lowest and most stable cancellations, closely shadowing the perfect-information benchmark. The robust approach remains slightly higher but still improves upon deterministic (expected) scheduling, while the deterministic (expected) policy performs moderately better than current practice. Overall, incorporating uncertainty into scheduling decisions substantially reduces emergency cancellations, approaching near-optimal performance even without exact foresight.

**Fig. 13:**
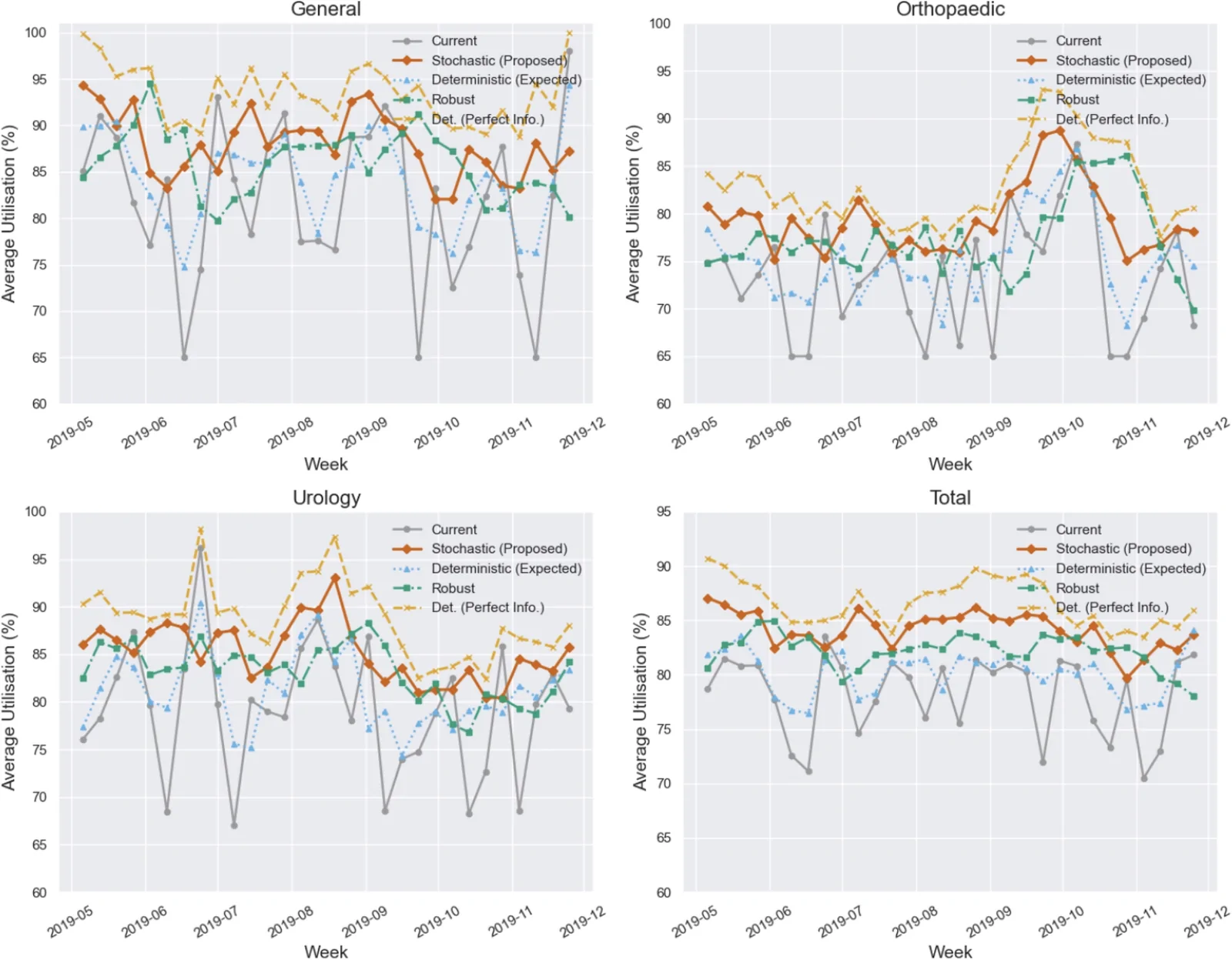
Time series of average utilisation by week (May to November 2019)

#### Average utilisation over time

Figure 13 presents the weekly average utilisation of ORs for each specialty under the five scheduling models. Overall, the two-stage stochastic model achieves the highest and most stable utilisation among the implementable approaches, while the perfect-information benchmark serves as an ideal upper bound. In contrast, the current system exhibits substantial variability due to unplanned disruptions and reactive scheduling.

For General Surgery, the current practice shows clear utilisation dips—particularly in June and September—signalling capacity underuse. Both deterministic (expected) and robust models mitigate these fluctuations, but the two-stage stochastic model delivers the highest and smoothest utilisation over time. The perfect-information line lies just above the stochastic trend, suggesting that the model captures nearly all attainable efficiency even without exact foresight. Occasional minor declines in the stochastic trend, such as in mid-September, may reflect weeks with fewer surgeries performed or temporary resource constraints.

In Orthopaedic Surgery, the current system again shows pronounced swings in utilisation, with deep troughs in June and September. The deterministic (expected) policy reduces some of these variations but remains affected by demand uncertainty. The robust policy maintains steadier but slightly conservative utilisation. The stochastic approach outperforms all others, sustaining high utilisation across nearly all weeks. Its close alignment with the perfect-information benchmark confirms the model’s capacity to approach optimal scheduling efficiency despite randomness in emergency arrivals.

For Urology, the current system demonstrates high variability with several utilisation drops around June and October. The deterministic (expected) model smooths some of these fluctuations, while the robust policy maintains greater stability but at a conservative level. The two-stage stochastic model achieves the highest and most stable utilisation, closely mirroring the perfect-information benchmark—indicating effective adjustment to fluctuating demand and case durations.

Across all specialties, the stochastic model achieves utilisation levels that are consistently close to the perfect-information optimum, outperforming the current, deterministic, and robust approaches. These findings demonstrate that explicitly modelling uncertainty not only enhances the stability of weekly utilisation but also enables near-optimal use of operating-room capacity under realistic, data-driven conditions.

### Sensitivity analysis

To examine the robustness of the proposed stochastic scheduling model, we conducted a set of one-way sensitivity analyses on key system parameters. These tests evaluate how changes in input assumptions influence major performance metrics, including total cost, emergency cancellations, and operating room utilisation.

To assess the effect of emergency demand on system performance, we vary the emergency arrival rate λ and report results separately for each specialty and weekday. Sensitivity analyses for surgery duration and cost parameters are instead aggregated by specialty, as these inputs exhibit stable distributions across weekdays. All experiments span the May–November 2019 horizon, with performance changes expressed as percentage deviations from the baseline stochastic solution.

#### Sensitivity to emergency arrival rate (λ)

For the emergency-arrival sensitivity experiment, we apply a 20% upward perturbation to the baseline arrival rate (λ) for every specialty and weekday. The reference rates ($\lambda _{0}$) correspond to the maximum-likelihood Poisson estimates for each specialty–weekday combination.

Table 7 summarises the resulting percentage changes in total cost, emergency cancellations, and average operating-room utilisation (OR utilisation is computed over regular hours only, overtime excluded).

Overall, the results indicate that a higher rate of emergency arrivals increases both total costs and cancellations across all specialties, with only a minor reduction in utilisation. Orthopaedic surgery shows the greatest sensitivity to increases in emergency demand, with total costs rising by up to 11% and emergency cancellations increasing by as much as 31% by the end of the week. This can be attributed to the specialty’s longer operation times and larger volume of patients on the elective waiting list, which reduce the system’s ability to accommodate sudden emergency inflows. In contrast, Urology shows the smallest deviation from baseline, indicating a more stable performance under demand uncertainty and greater scheduling flexibility.

**Table 7:** Sensitivity of the two-stage stochastic model to a 20% increase in emergency arrival rate (λ) across specialties and weekdays, relative to baseline ($\lambda _0$)

Specialty	Day	Δ Cost (%)	Δ Canc. (%)	Δ Util. (pp)
*General*	Mon	+7.8	+22.5	-1.2
	Tue	+6.5	+20.1	-1.1
	Wed	+7.1	+21.3	-1.2
	Thu	+8.7	+23.9	-1.4
	Fri	+9.3	+26.4	-1.7
*Orthopaedic*	Mon	+9.0	+27.8	-1.5
	Tue	+8.1	+25.4	-1.4
	Wed	+9.7	+28.9	-1.6
	Thu	+10.5	+30.1	-1.8
	Fri	+11.4	+31.2	-1.9
*Urology*	Mon	+5.9	+18.7	-1.0
	Tue	+5.6	+17.9	-0.9
	Wed	+6.1	+19.5	-1.0
	Thu	+6.8	+20.3	-1.1
	Fri	+7.3	+21.5	-1.3

*Note.* Baseline rate ($\lambda _0$) is the MLE from a Poisson model fitted to emergency arrivals for each specialty and weekday. The scenario applies a 20% increase ($1.2\lambda _0$) while all other parameters are held fixed

**Table 8:** Sensitivity of the two-stage stochastic model to lognormal surgery-duration parameters by specialty

Scenario	Specialty	Δ Cost (%)	Δ Canc. (%)	Δ Util. (pp)
*(i) * $\mu {+}\ln (1.10)$ * — All durations scaled by * +10%				
	General	+10.3	+8.6	-1.9
	Orthopaedic	+11.1	+9.3	-2.0
	Urology	+8.5	+6.7	-1.4
*(ii) * $\mu {-}\ln (1.10)$ * — All durations scaled by * -10%				
	General	-9.2	-7.3	+1.7
	Orthopaedic	-10.2	-8.1	+1.8
	Urology	-7.8	-5.9	+1.2
*(iii) * $\sigma {\times }1.20$ * — Dispersion increased by * +20% * (median fixed)*				
	General	+4.1	+9.8	-0.5
	Orthopaedic	+4.2	+12.1	-0.6
	Urology	+3.6	+8.9	-0.4

*Note.* Results shown relative to fitted baseline $(\mu _0,\sigma _0)$

**Table 9:** Sensitivity of the stochastic (SAA) model to a 20% increase in cost parameters by specialty

Specialty	Parameter	Δ Cost (%)	Δ Canc. (%)	Δ Util. (pp)
*General*	$C^a_{ib}$	+2.1	-1.2	-0.2
	$C^a_{ib'}$	+1.4	-0.8	-0.1
	$C^c_{i}$	+5.9	-6.7	-0.3
	$C^a_{j}$	+3.6	-1.1	-0.2
	$C^c_{j}$	+7.8	-9.4	-0.5
	$C^o_{b}$	+2.9	+0.7	-0.1
*Orthopaedic*	$C^a_{ib}$	+2.3	-1.3	-0.2
	$C^a_{ib'}$	+1.6	-0.9	-0.1
	$C^c_{i}$	+6.1	-7.1	-0.4
	$C^a_{j}$	+3.8	-1.2	-0.2
	$C^c_{j}$	+8.3	-9.9	-0.5
	$C^o_{b}$	+3.2	+0.8	-0.1
*Urology*	$C^a_{ib}$	+1.9	-1.0	-0.2
	$C^a_{ib'}$	+1.3	-0.7	-0.1
	$C^c_{i}$	+5.4	-6.0	-0.3
	$C^a_{j}$	+3.1	-0.9	-0.2
	$C^c_{j}$	+7.0	-8.7	-0.4
	$C^o_{b}$	+2.7	+0.6	-0.1

*Note.* $C^a_{ib}$: elective allocation; $C^a_{ib'}$: postponement; $C^c_{i}$: elective cancellation; $C^a_{j}$: emergency allocation; $C^c_{j}$: emergency cancellation; $C^o_{b}$: overtime. Results shown as percentage deviation from baseline outcomes

#### Sensitivity to surgery duration parameters (Lognormal Model)

Surgery durations were assumed to follow a lognormal distribution, where the natural logarithm of duration is normally distributed as $\ln (D)\sim \mathcal {N}(\mu _0,\sigma _0^{2})$. The parameters $(\mu _0,\sigma _0)$ were estimated based on synthetic data from May–Nov 2019 for each specialty. In this model, μ determines the typical (central) duration, and σ controls how much variation there is among surgeries. The median duration is given by $\exp (\mu )$, while the mean duration equals $\exp \!\big (\mu +\tfrac{1}{2}\sigma ^{2}\big )$. The baseline scenario uses the maximum-likelihood estimates $(\mu _0,\sigma _0)$ obtained from the fitted lognormal distribution for each specialty.

To evaluate robustness against estimation uncertainty, we examined three controlled changes in the lognormal parameters relative to the stochastic (SAA) baseline: (i) $\mu \leftarrow \mu _0 + \ln (1.10)$, which increases all typical surgery durations (including the mean and median) by about 10%; (ii) $\mu \leftarrow \mu _0 - \ln (1.10)$, which decreases all durations by about 10%; and (iii) $\sigma \leftarrow 1.20\,\sigma _0$ while keeping $\mu =\mu _0$, which makes durations more variable by 20%—producing a wider spread between short and long cases without changing the median value.

Table 8 reports percentage changes in total cost and cancellations, and percentage-point (pp) changes in average OR utilisation relative to baseline.

Increasing μ (shifting all durations upward) multiplicatively raises the expected duration $\mathbb {E}[D]$, so cases run longer on average—costs rise, emergency cancellations increase, and regular-hours utilisation falls slightly as fewer, longer surgeries occupy the same time. Decreasing μ produces the opposite effect.

Raising dispersion (σ) by 20% widens the spread of surgery durations while keeping the median unchanged. This added variability makes schedules less predictable: some cases finish early, but others run far longer than expected. The resulting uncertainty causes modest cost growth but consistently higher cancellations due to overruns and schedule conflicts.

The Orthopaedic specialty is the most sensitive, likely because of its longer baseline operations and heavier elective queues, so even small increases in duration or variability spill into overtime and displace emergency cases. Urology, with shorter and more homogeneous procedures, is correspondingly more robust.

#### Sensitivity to cost parameters

This analysis examines how changes in cost coefficients influence system performance and the overall objective value. Each parameter was increased by 20% from its baseline value (see Table 4), while all other parameters remained unchanged. The model was then re-optimised under each modified cost setting. Reported metrics show percentage changes in total objective cost and cancellations, and percentage-point (pp) changes in average OR utilisation (Table 9). Therefore, the values reported in the “Δ Cost (%)” column represent the percentage deviation in the total objective value, rather than the isolated change in the corresponding cost component alone. Since the objective function includes allocation, postponement, cancellation, and overtime costs, increasing a single cost coefficient may still increase the total objective value even when the model adapts the schedule to reduce cancellations, overtime, or inefficient OR utilisation.

Emergency- and cancellation-related costs have the greatest impact on overall system behaviour. Among specialties, Orthopaedics—due to its longer procedures and tighter waiting list—shows the strongest overall response to these cost increases.

The system is most sensitive to the costs linked with emergency and elective cancellations, meaning that penalties for unserved cases largely determine overall performance. Orthopaedics is affected the most—its long procedures and compressed operating lists make it vulnerable to even small cost changes, which quickly raise total costs and cancellations. Urology, on the other hand, stays fairly steady because its surgeries are shorter and more predictable.

## Conclusion

This study presents a two-stage stochastic model designed to optimise the scheduling of elective and emergency surgeries within hospital ORs, addressing the complexities and uncertainties inherent in surgery durations and emergency case arrivals. By incorporating flexible scheduling with buffer times for emergencies, the model offers a robust solution for enhancing hospital operational efficiency.

The proposed model was solved using the Sample Average Approximation (SAA) method to capture the stochastic nature of uncertainty through multiple simulated scenarios. To benchmark performance, a robust (min–max) optimisation model—inspired by the approach of Neyshabouri and Berg [4]—was developed using the Column-and-Constraint Generation (CCG) algorithm to represent worst-case decision-making. These were compared against deterministic and perfect-information benchmarks to evaluate trade-offs between performance, risk aversion, and computational efficiency.

Using a synthetic dataset calibrated to an Australian hospital’s operating theatre (May–November 2019) across General, Orthopaedic, and Urology specialties, the results confirm the superiority of the stochastic framework. The model achieves significant improvements across key performance metrics, including reduced total costs, increased utilisation, and fewer emergency cancellations, while maintaining practical computation times. Notably, it consistently outperforms deterministic and robust benchmarks, producing results close to the perfect-information upper bound—confirming its balance between realism and efficiency. These findings also underscore the model’s capacity to adapt to fluctuating demands and prioritise urgent cases while maintaining overall system stability.

Several key insights emerge from the analysis. First, the model slightly reduces the number of elective surgeries to reserve capacity for emergency arrivals, achieving a strategic balance between planned and unplanned cases without major loss in elective throughput. Second, a noticeable increase in performed emergency cases demonstrates the model’s responsiveness and ability to reduce cancellations, ensuring urgent cases are prioritised effectively. Third, average OR utilisation improved from approximately 78% to 84%, indicating more efficient use of hospital resources through proactive buffer allocation. Fourth, the model reduced total costs by about 18% across all specialties, with the most substantial gains observed in orthopaedics—where high variability and longer durations typically create scheduling bottlenecks. Finally, despite its size and stochastic formulation, the SAA-based model remains computationally tractable, with runtime only about twice that of deterministic models and also faster than the conservative robust counterpart. This confirms its suitability for real-world hospital implementation.

In conclusion, the integration of stochastic optimisation, flexible buffer placement, and dynamic OR allocation proves highly effective in enhancing the responsiveness and resilience of surgical scheduling systems. The model not only improves operational metrics but also provides a transparent decision-making framework that supports planning under uncertainty.

Despite these promising results, several limitations should be acknowledged. First, the model is evaluated using data from a single Australian hospital, which may limit the direct generalisability of the findings to other settings. Second, other key operational constraints—such as detailed staffing rosters, anaesthetist availability, equipment limitations, and downstream capacity (for example, PACU or ICU beds)—are not explicitly modelled, even though they can influence the feasibility of schedules.

Future research may extend this work by explicitly modelling additional resource constraints (for example, staff and equipment availability) and validating the model across multiple hospitals.

Overall, this study reinforces the value of uncertainty-aware decision-making in hospital operations and provides a foundation for practical, efficient, and adaptable OR scheduling models in real healthcare environments.

## Figures and Tables

**Fig. 14:**
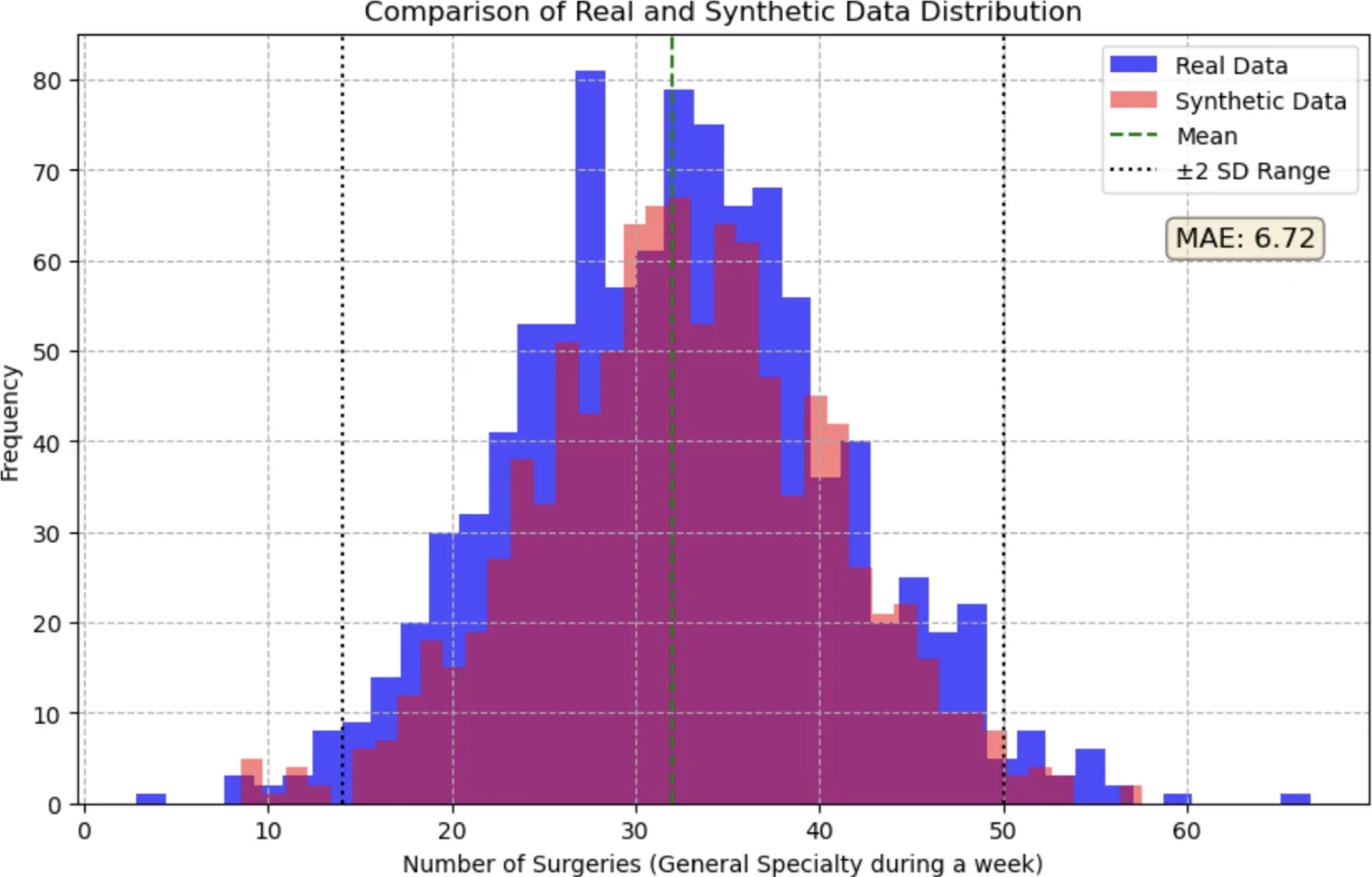
Example of the generated synthetic data

**Fig. 15:**
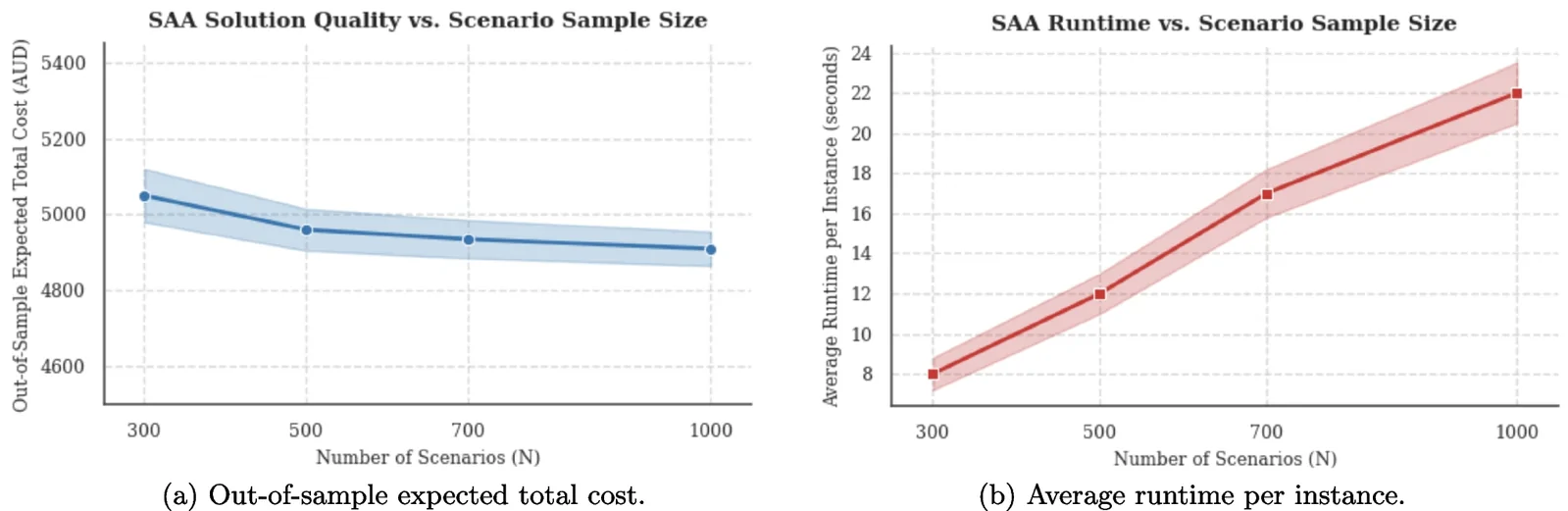
Convergence of solution quality (left) and runtime (right) with increasing number of scenarios *N* in the SAA

## Data Availability

The datasets generated during and/or analysed during the current study are available from the corresponding author on reasonable request.

## A Synthetic dataset

In this study, we generated a synthetic dataset based on real hospital data to ensure confidentiality while maintaining the statistical characteristics necessary for valid analysis. The following steps outline the data synthesis process:

1. **Determine probability distribution:** Using historical hospital data, key statistical parameters were derived to model the distribution of surgical cases. For example, the number of weekly elective surgeries in the General specialty follows a normal distribution with a mean (μ) of 32 and a standard deviation (σ) of 9, represented as $\mathcal {N}(\mu = 32, \sigma ^2 = 81)$ (Fig. 14). This distribution ensures that the generated synthetic data closely mimics real-world patterns while adhering to privacy requirements. Also, in line with the Law of Large Numbers, increasing the sample size of the generated scenarios leads to synthetic data that converges more closely to the statistical properties of the real dataset
2. **Generate synthetic data:** Using the defined distribution parameters, synthetic values were generated to replicate the statistical properties of the real-world dataset. This approach produces realistic yet anonymised data points for each specialty, maintaining similar trends and variability as the original hospital data.
3. **Validate synthetic data:** To ensure the validity and reliability of the synthetic dataset, it was compared with the real dataset using error metrics such as Mean Absolute Error (MAE) and Root Mean Squared Error (RMSE). These measures quantify the similarity between synthetic and real data distributions, confirming that the generated dataset adequately represents the hospital’s operational patterns (Table 10).

**Table 10:** Error metrics comparing real and synthetic datasets across specialties (May-November 2019)

Specialty	MAE (Elective)	RMSE (Elective)	MAE (Emergency)	RMSE (Emergency)
General	2.8	3.6	0.9	1.3
orthopaedics	3.5	4.2	1.1	1.5
Urology	1.9	2.4	0.7	1.1

The error metrics, computed over the six-month period from May to November 2019, indicate that the synthetic data closely approximates the real dataset. This confirms that the synthesised dataset captures the essential operational characteristics required for model evaluation. Overall, the synthetic dataset provides a robust and confidential foundation for analysis and testing within the proposed model, ensuring that all numerical results remain statistically representative without disclosing sensitive hospital information.

## B Scenario size justification for the SAA approach

To justify the choice of N=500 scenarios in the Sample Average Approximation (SAA), we conducted a numerical convergence study on a subset of representative daily scheduling instances drawn from the May–November 2019 horizon across three specialties (orthopaedics, General, and Urology). For each instance, the two-stage stochastic model was solved using different scenario sample sizes, $N \in \{300, 500, 700, 1000\}$, with multiple independent replications for each *N*.

Two criteria were used to evaluate convergence:

- **Solution quality.** The first-stage schedule obtained for each *N* was evaluated on a large out-of-sample validation set to estimate its expected total cost. As shown in Fig. 15a, the out-of-sample cost decreases as *N* increases but stabilises beyond N=500, indicating that additional scenarios yield only marginal improvements in accuracy.
- **Computational efficiency.** The average runtime per instance grows approximately linearly with *N*, as illustrated in Fig. 15b. Beyond N=500, the gain in solution quality is negligible compared with the increase in computational effort.

Based on this trade-off between accuracy and runtime, we adopted N=500 as a balanced and robust scenario size for all 540 daily problem instances. This choice ensures stable objective estimates and consistent scheduling decisions while maintaining computational feasibility for large-scale hospital optimisation studies.

## Code Availability

The code used for data analysis in this study is available from the corresponding author on reasonable request.
